# Lipoproteins in *Drosophila melanogaster*—Assembly, Function, and Influence on Tissue Lipid Composition

**DOI:** 10.1371/journal.pgen.1002828

**Published:** 2012-07-26

**Authors:** Wilhelm Palm, Julio L. Sampaio, Marko Brankatschk, Maria Carvalho, Ali Mahmoud, Andrej Shevchenko, Suzanne Eaton

**Affiliations:** Max Planck Institute of Molecular Cell Biology and Genetics, Dresden, Germany; Max Planck Institute for Biophysical Chemistry, Germany

## Abstract

Interorgan lipid transport occurs via lipoproteins, and altered lipoprotein levels correlate with metabolic disease. However, precisely how lipoproteins affect tissue lipid composition has not been comprehensively analyzed. Here, we identify the major lipoproteins of *Drosophila melanogaster* and use genetics and mass spectrometry to study their assembly, interorgan trafficking, and influence on tissue lipids. The apoB-family lipoprotein Lipophorin (Lpp) is the major hemolymph lipid carrier. It is produced as a phospholipid-rich particle by the fat body, and its secretion requires Microsomal Triglyceride Transfer Protein (MTP). Lpp acquires sterols and most diacylglycerol (DAG) at the gut via Lipid Transfer Particle (LTP), another fat body-derived apoB-family lipoprotein. The gut, like the fat body, is a lipogenic organ, incorporating both *de novo*–synthesized and dietary fatty acids into DAG for export. We identify distinct requirements for LTP and Lpp-dependent lipid mobilization in contributing to the neutral and polar lipid composition of the brain and wing imaginal disc. These studies define major routes of interorgan lipid transport in *Drosophila* and uncover surprising tissue-specific differences in lipoprotein lipid utilization.

## Author Summary

Lipoproteins transport both dietary and endogenously synthesized lipids between different organs. Lipoprotein dysfunction is associated with many medical disorders, including cardiovascular disease, but the mechanisms underlying pathogenesis are unclear. Simple animal models would be valuable, therefore, to understand basic functions of lipoprotein and their influence on tissue lipids. Here, we develop the fruit fly *Drosophila melanogaster* as a genetically tractable model to study lipoprotein metabolism. We characterize the major *Drosophila* lipoproteins, the lipids they transport through circulation, and the mechanisms by which they acquire lipid cargo from different organs. By genetically blocking specific inter-organ lipid transport routes, we uncover surprising tissue-specific differences in lipoprotein lipid utilization. Lipoproteins deliver lipids from fat body and gut to wing imaginal discs, which utilize them to build their fat stores. In addition, lipoproteins provide a significant fraction of membrane phospholipids to wing disc and gut cells. In contrast, fat storage in the brain does not require lipoprotein-mediated delivery of lipids from the gut, and the brain phospholipid composition can be maintained independently of lipoproteins. Our studies define basic features of *Drosophila* lipoprotein metabolism and suggest novel mechanisms for how lipoproteins might affect animal tissue function in general.

## Introduction

Lipoproteins allow the transport of lipids between different organs. In humans, perturbed lipoprotein levels correlate with metabolic disease, but to which extent they contribute to tissue pathology is unclear. Animals synthesize a huge variety of lipids that form cellular membranes, function as signaling molecules, and constitute the major storage and transport form of energy. The lipid composition of different cell types and tissues is important for biological function. To what extent do lipoproteins influence these cellular properties?

Mammals have two types of apolipoproteins that scaffold particles with different functions [1]. Several proteins of the exchangeable apolipoprotein family, including apoA-I, scaffold high-density lipoproteins (HDL), which mediate reverse cholesterol transport. ApoB scaffolds very low-density lipoproteins (VLDL) and chylomicrons, which are secreted by the liver and gut, and deliver fat and sterols to peripheral tissues. Mammalian apoB acquires lipid in producing cells by a process requiring MTP [2], [3]. In humans, MTP deficiency blocks secretion of apoB-containing lipoproteins, resulting in abetalipoproteinemia [4]. This causes fatty liver, intestinal lipid malabsorption, and defects in peripheral tissue function including ataxia, retinal degeneration and anemia [5]. On the other hand, elevated levels of apoB-containing lipoproteins are a hallmark of metabolic syndrome, a pathological condition comprising wide-ranging dysfunctions in different tissues. These include obesity, diabetes, heart disease and increased risk of dementia [6], [7]. Mammalian tissue culture cells preferentially derive fatty acids and cholesterols from lipoproteins, but can switch to endogenous synthesis if lipoproteins are not provided [8], [9]. However, it is not clear to what extent autonomous synthesis suffices for different tissues to maintain a normal lipidome *in vivo*. Furthermore, while the influence of dyslipidemia on the plasma lipidome has been well studied, less attention has been paid to organism-wide changes in tissue lipid composition. Advances in lipid mass spectrometry are only beginning to make such studies possible [10].

To investigate how lipoproteins influence tissue lipid composition requires a system where lipoproteins can be manipulated in a time and tissue-dependent manner. *Drosophila* genetics could provide a tool to easily control lipoprotein levels in an organism whose metabolism shares many similarities with that of mammals [11], [12]. In *Drosophila*, the molecular mechanisms controlling storage and mobilization of neutral lipid in cellular lipid droplets resemble mammalian pathways [13], [14]. This similarity even extends to the progression of metabolic diseases caused by dysfunctions in lipid metabolism [15]. The major lipoproteins of *Drosophila* and other insects, the lipophorins (Lpp), are similar to mammalian apoB-containing lipoproteins; their scaffolding apolipoproteins, the apolipophorins (apoLpp), are members of the apoB family, which is conserved throughout the animal kingdom [16]. Moreover, *Drosophila* lipoprotein receptors resemble those of mammals [17]. The low-density lipoprotein (LDL) receptor homologues LpR1 and LpR2 promote Lpp internalization [18], but also appear to increase cellular neutral lipid storage by non-endocytic mechanisms [19]. Similarly, the role of heparan sulfate proteoglycans as endocytic lipoprotein receptors is conserved between mammals and flies [20], [21].

Insect lipoproteins have been best studied in *Manduca sexta* and *Locusta migratoria*, large insects amenable to biochemical and physiological manipulations [22], [23]. It has been proposed that insects produce Lpp exclusively in the fat body [24], which functions analogously to mammalian liver and white adipose tissue [25]. However, Lpp can take up neutral lipid from both fat body and gut when added externally to explanted tissues. This lipidation mechanism requires activity of another lipoprotein, Lipid Transfer Particle (LTP) [26], [27], [28], and appears to differ from the MTP-dependent lipidation of mammalian apoB in the secretory pathway of producing cells.

In *Drosophila*, initial studies have shown that Lpp knock-down in the fat body causes accumulation of neutral lipid in the gut, providing *in vivo* support for models developed from physiological experiments in other insects [29]. However, whether *Drosophila* might produce particles similar to the LTP of other insects has not been addressed, because the gene(s) encoding insect LTP apolipoproteins have been unknown. The *Drosophila* genome does encode a homologue of MTP, and this protein facilitates secretion of mammalian apoB and locust apoLpp in heterologous tissue culture systems [30], [31]. This raises the possibility that *Drosophila* Lpp assembly might resemble that of mammalian apoB-containing lipoproteins, but the requirement for MTP has never been examined *in vivo*.

Here, we characterize lipoprotein function in *Drosophila*. We identify three circulating lipoproteins in *Drosophila* larvae, and analyze their source, functions, and mechanisms of secretion and lipid loading. The development of high-resolution shotgun lipidomics for the first time allows the precise and comprehensive quantification of many different lipid species with a sensitivity that makes it possible to study individual *Drosophila* tissue lipidomes. Therefore, we have combined the power of *Drosophila* genetics with mass spectrometry to investigate systematically and quantitatively how lipoproteins influence tissue lipid composition.

## Results

### *Drosophila* Possesses Two Homologues of ApoB, but No ApoA

**Figure 1:**
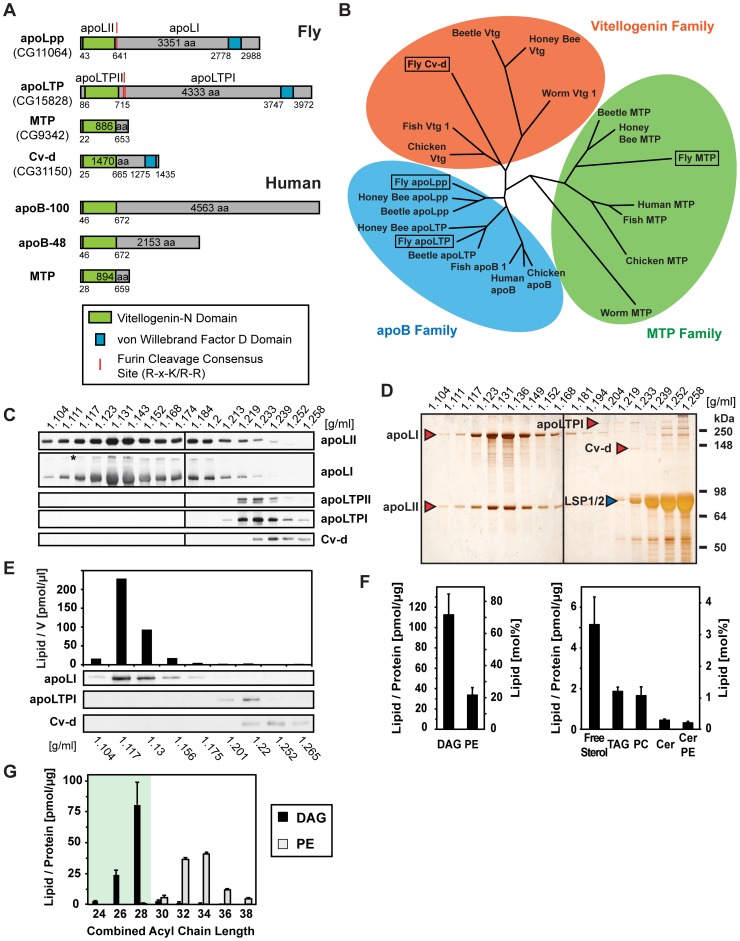
*Drosophila* lipoproteins and their lipid content. (A) Fly and human proteins harboring vitellogenin-N domains. *Drosophila* possesses two apoB homologues, apoLpp and apoLTP, an MTP and a vitellogenin-like protein, Cv-d. Humans possess one apoB gene, which gives rise to two proteins, apoB-100 and apoB-48, and an MTP. Both apoLpp and apoLTP give raise to two polypeptides. The N-terminal and C-terminal parts generated from full-length apoLpp are denoted apoLII and apoLI, respectively. The N-terminal and C-terminal parts generated from full-length apoLTP are denoted apoLTPII and apoLTPI, respectively. Numbers in the protein boxes denote the predicted total number of amino acid (aa); numbers below the boxes indicate the first and last amino acid of the predicted vitellogenin-N and von Willebrand factor D domains. (B) Phylogenetic tree of animal vitellogenin-N domain proteins. Alignment and tree were constructed from vitellogenin-N domain sequences with ClustalW and PHYLIP-NEIGHBOR using http://toolkit.tuebingen.mpg.de and default settings. See also [16]. (C) Immunoblot of larval 3rd instar hemolymph lipoproteins fractionated on an isopycnic KBr gradient. ApoLI and apoLII scaffold the lipoprotein Lpp; apoLTPII and apoLTPI scaffold the lipoprotein LTP. Note that minor amounts of uncleaved apoLpp (*) are present in the hemolymph. (D) Silver staining of an isopycnic gradient run and processed in parallel to the gradient shown in (C). Silver-stained bands corresponding to immunoblot bands in (C) are indicated by red arrowheads. Note that the non-lipidated larval serum proteins (LSP) 1 and 2 (blue arrowhead) peak in the last gradient fraction. (E) Larval hemolymph fractionated on an isopycnic KBr density gradient, analyzed by mass spectrometry and immunoblotting. Shown is the total amount of lipid present in each gradient fraction from 1 µl hemolymph of one representative experiment. More than 95% of all hemolymph lipids co-fractionate with Lpp. (F) Lipid composition of the larval hemolymph, quantified by mass spectrometry (n = 8, free sterols n = 4). The left y-axes indicate the amount of each lipid class normalized to total hemolymph protein, the right y-axes the mol% of each lipid class. (G) Chain length distribution of fatty acid residues in DAG and PE in larval hemolymph, quantified by mass spectrometry (n = 8). The amount of each lipid species is normalized to total hemolymph protein. Lpp DAG contains fatty acid residues with an average chain length of 12–14 carbons (medium-chain); Lpp PE contains fatty acid residues with an average chain length of 16–18 carbons (long-chain). For all mass spectrometry data, lipid species with medium-chain fatty acid residues are indicated by green background; error bars indicate ±SD (* p<0.05, ** p<0.005, *** p<0.0001).

We started our study of *Drosophila* lipoprotein metabolism with a genome search for potential apolipoproteins. Many proteins involved in interorgan lipid transport harbor vitellogenin-N domains, including apoB, MTP and vitellogenins [16], [32], [33]. BLAST searches with the vitellogenin-N domain of human apoB yield four fly genes: *apolpp* [34] and *mtp* [31], as well as two novel genes, CG15828 and CG31150 (Figure 1A). *apolpp* and CG15828 seem to have arisen by gene duplication of an ancestral insect apoB homologue (Figure 1B). As will be shown below, the protein encoded by CG15828 assembles a lipoprotein that functions similarly to a lipid transfer particle, LTP, identified in *Locusta* and *Manduca* [35], [36]. We therefore refer to it as apoLTP. CG31150, which has been recently shown to be mutated in *crossveinless d* (*cv-d*) [37], is most closely related to vitellogenins (Figure 1B).

To ask whether the fly genome encoded exchangeable apolipoproteins like those scaffolding mammalian HDL, we searched for sequences similar to apolipoproteins A-I and E. No *Drosophila* protein had significant homology. Neither was there a homologue of apoLipophorin III, a structurally related exchangeable apolipoprotein in *Locusta* and *Manduca* [38]. Thus, there is no evidence for apolipoproteins of this family in *Drosophila*.

### Drosophila Larvae Have Three Circulating Lipoproteins of Different Densities—Lpp, LTP, and Cv-d

To ask which of the vitellogenin-N domain proteins might scaffold lipoproteins, we fractionated hemolymph from feeding third instar larvae in isopycnic gradients and probed for apoLpp, apoLTP and Cv-d. These proteins are all present in circulation (Figure S1A), and fractionate at densities consistent with different degrees of lipidation (Figure 1C). ApoLpp is posttranslationally cleaved into apoLII and apoLI, which assemble the lipoprotein Lpp (1.13–1.14 mg/ml). Like apoLpp, apoLTP harbors furin cleavage sequences C-terminal to the vitellogenin-N domain, and is cleaved into two polypeptides, which we denote apoLTPII and apoLTPI. ApoLTPII and apoLTPI assemble a higher density (1.23 mg/ml) lipoprotein, LTP. Cv-d is poorly lipidated (1.24 mg/ml), consistent with its similarity to vitellogenins, which contain little lipid [39].

To investigate the relative amounts of Lpp, LTP and Cv-d in circulation, we used silver and Coomassie staining to detect them in hemolymph fractionated by density and size (Figure 1D, Figure S1B). These methods detect two prominent bands corresponding to apoLII and apoLI. ApoLTPI and Cv-d are detectable, but much less abundant. Each of these proteins is also present in embryos and adults (Figure S1C). We do not detect any other proteins in low-density fractions, suggesting that no other abundant lipoproteins exist in larval hemolymph.

### Lipophorin Is the Major Hemolymph Lipid Carrier

To assess the amount of lipid associated with each lipoprotein, we quantified hemolymph lipids in different density fractions by shotgun mass spectrometry. More than 95% of hemolymph lipids co-fractionate with Lpp (Figure 1E, Figure S1D). The fractions containing LTP and Cv-d account for less then 1% and 0.5% of hemolymph lipids, respectively. Thus, Lpp carries the bulk of lipids in circulation.

The most abundant Lpp lipids are diacylglycerol (DAG) (70 mol%) and phosphatidylethanolamine (PE) (20 mol%) (Figure 1F). Sterols comprise 5 mol% of Lpp lipids; the remainder includes triacylglycerol (TAG), phosphatidylcholine (PC), ceramide (Cer) and ceramide phosphorylethanolamine (CerPE). More than 95% of Lpp DAG species have a combined acyl chain length of only 26 or 28 carbons, suggesting they contain mostly medium-chain fatty acid residues (12 or 14 carbons) (Figure 1G). This differs strikingly from Lpp phospholipids, which have a combined acyl chain length of 32 to 36 carbons, suggesting they contain long-chain fatty acid residues (16 or 18 carbons).

It was more difficult to assess the lipid composition of the low-abundant LTP and Cv-d. However, we noted that fractions containing LTP are relatively enriched in sphingolipids with hydroxylated fatty acids (Figure S1E).

### Lpp and LTP Originate in the Fat Body and Depend on MTP for Their Production

Mammalian apoB-containing lipoproteins are secreted from both liver and gut. While the insect fat body similarly secretes Lpp, the insect gut does not appear to produce Lpp [23], [24]. To ask which of the different *Drosophila* lipoproteins were produced by the fat body or gut, we used reverse transcription PCR to determine the presence of *apolpp*, *apoltp*, and *cv-d* transcripts in these organs. *apolpp*, *apoltp*, and *cv-d* transcripts are readily detectable in the fat body (Figure S2). In contrast, none of them can be detected in the gut. Thus, the *Drosophila* larval gut does not produce any of these lipoproteins.

**Figure 2:**
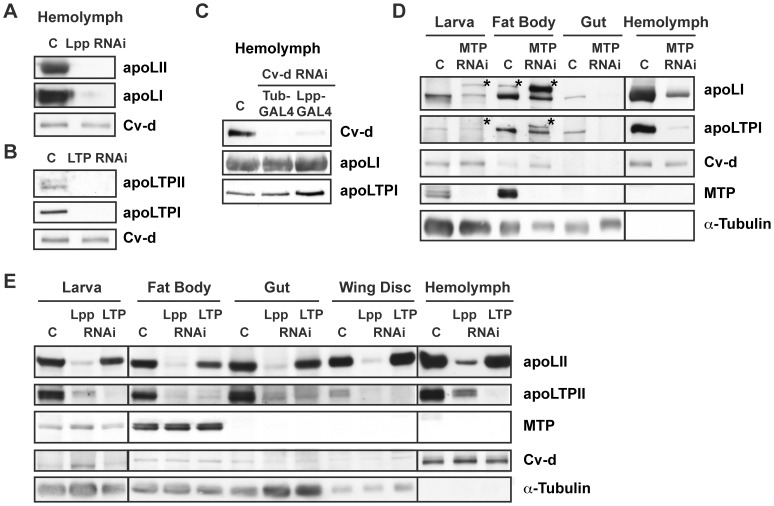
Lpp and LTP originate from the fat body and depend on MTP for their secretion. (A–C) Immunoblots of hemolymph from larvae in which (A) Lpp, (B) LTP, or (C) Cv-d is specifically knocked down in the fat body. Fat body-specific RNAi strongly decreases lipoprotein levels in the hemolymph. Note that ubiquitous knock-down with Tub-GAL4 depletes Cv-d even further, suggesting some contribution of other organs. (D) Immunoblot of organs and hemolymph from MTP RNAi larvae, showing that secretion of Lpp and LTP is strongly reduced upon knock-down of MTP. (*) indicates the uncleaved full-length precursors apoLpp and apoLTP, which accumulate in the fat body of MTP RNAi larvae. (E) Immunoblot of larval hemolymph and organs from Lpp or LTP RNAi larvae.

To ask what fraction of circulating Lpp, LTP and Cv-d was derived from the fat body, we blocked their production in this tissue by RNAi. Fat body-specific knock-down strongly reduces levels of Lpp, LTP and Cv-d in the hemolymph, establishing this organ as the major source of circulating lipoproteins (Figure 2A–2C).

*mtp* transcripts are readily detectable in the fat body, similar to what we observed for transcripts of the different apolipoproteins (Figure S2). To ask whether production of Lpp, LTP or Cv-d depended on MTP, we knocked down MTP in the fat body by RNAi and examined hemolymph lipoproteins. MTP RNAi causes the buildup of the uncleaved full-length apolipoprotein precursors apoLpp and apoLTP in the fat body, and strongly reduces hemolymph Lpp and LTP levels (Figure 2D). Thus, *Drosophila* MTP has a conserved function in the production of apoB-family lipoproteins *in vivo*. However, MTP RNAi does not reduce levels of Cv-d in circulation; thus, not all proteins with vitellogenin-N domains depend on MTP for their release to circulation. These data distinguish the vitellogenin-like lipoprotein Cv-d from canonical vitellogenins in other organisms, whose release is promoted by MTP [40], [41].

### Lpp and LTP Function Together to Mobilize Lipids from the Gut

**Figure 3:**
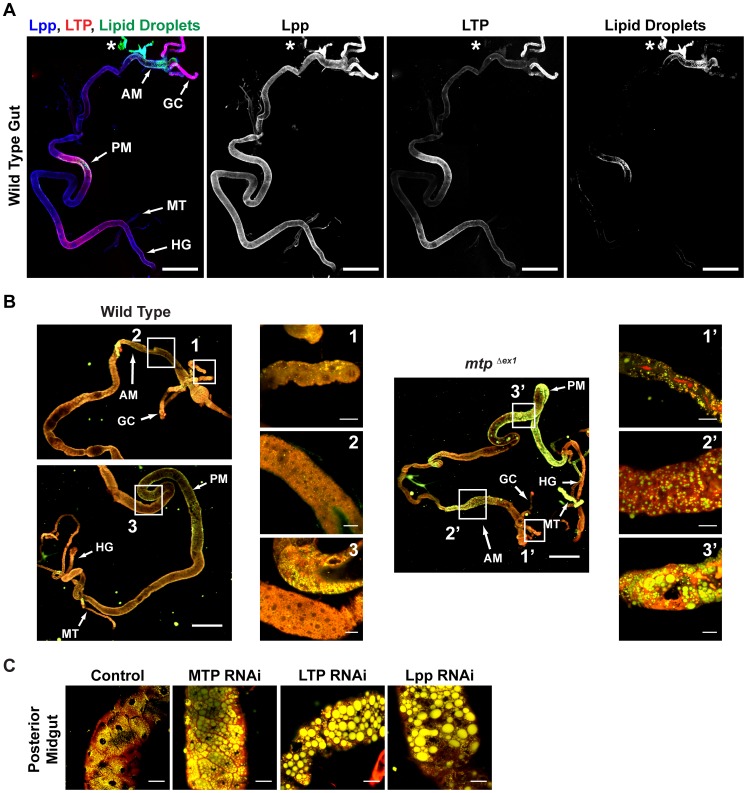
Lpp and LTP function together to mobilize lipids from the gut. (A) Immunofluorescence of a second instar larval gut, stained for Lpp, LTP and neutral lipid droplets. Lpp, LTP and lipid droplets are enriched in the same subsets of the anterior and posterior midgut. (*) indicates a nearby piece of fat body. GC: gastric caeca; AM: anterior midgut; PM: posterior midgut; HG: hindgut; MT: malpighian tubule. Scale bars = 200 µm. (B) Lipid droplets of guts from first instar wild-type and *mtp^Δex1^* larvae, visualized with Nile red. Loss of MTP causes strong neutral lipid accumulation. Yellow: neutral lipids; red: phospholipids. GC: gastric caeca; AM: anterior midgut; PM: posterior midgut; HG: hindgut. Scale bars = 200 µm. Scale bars blow ups = 20 µm. (C) Lipid droplets of posterior midguts upon MTP, LTP or Lpp RNAi, visualized with Nile red. Knock-down of either protein causes similar accumulation of neutral lipid. Scale bars = 50 µm.

Although Lpp originates in the fat body, we previously showed that its knock-down causes the buildup of neutral lipid in the gut [29]. To ask whether Lpp, or other lipoproteins, are recruited to the gut, we assessed lipoprotein levels in this organ by Western blotting. These experiments show that Lpp and LTP are readily detectable in the gut (Figure 2E). LTP is most abundant in gastric caecae, and in subsets of the anterior and posterior midgut (Figure 3A). Lpp has a broader distribution, but is enriched in the same regions. Interestingly, neutral lipid droplets are most abundant in these same regions of the anterior and posterior midgut, suggesting they may be involved in dietary lipid mobilization. To confirm that LTP and Lpp in the gut are derived from the fat body, we blocked Lpp and LTP secretion from the fat body using tissue-specific MTP RNAi. This strongly reduces their levels in the gut (Figure 2D). Thus, Lpp and LTP produced in the fat body enter circulation and are recruited to the gut.

To ask whether either Lpp or LTP were required to export lipid from the gut to circulation, we studied *mtp* mutant larvae, which arrest in the first larval instar and do not secrete these lipoproteins (Figure S3A–S3D). Loss of MTP increases the size and number of neutral lipid droplets in the anterior and posterior midgut and, more moderately, in the gastric caecae (Figure 3B). We wondered whether lipoprotein production in the fat body would be sufficient for the mobilization of lipids from the gut. To investigate this, we restored MTP activity specifically in the fat body of *mtp* mutant larvae (Figure S3E). This reduces size and number of lipid droplets in the midgut to wild-type levels, indicating that Lpp and LTP production in the fat body suffices to effect lipid export from the gut (Figure S3F).

To investigate individual requirements for Lpp and LTP for lipid export from the gut, we blocked their production by mutation, or by RNAi-mediated knock-down of the respective apolipoprotein. *apolpp* mutants die as embryos; *apolpp* is transcribed in the embryonic yolk (Figure S3G) [34], suggesting that embryonic lethality might result from a failure to mobilize maternal lipid stores. Initiating Lpp RNAi during larval stages reduces Lpp levels by more than 90% (Figure S3H) and dramatically enlarges neutral lipid droplets in the gut (Figure 3C). Interestingly, expression of two independent Lpp RNAi constructs also reduces LTP levels in the hemolymph and in all organs, including the fat body (Figure 2E, Figure S3H, and data not shown). Thus, LTP production and/or turnover may be influenced by Lpp.

Similar to *mtp* mutants, *apoltp* mutants arrest in early larval development (Figure S3I–S3L). Surprisingly, although LTP carries such a small proportion of hemolymph lipids, *apoltp* mutant larvae also accumulate large neutral lipid droplets in the gut (Figure S3M). The lipid accumulation caused by loss of LTP appears identical to that caused by loss of MTP or Lpp. RNAi-mediated knock-down of LTP in the fat body reduces levels of LTP by more than 95% (Figure S3H) and causes a gut phenotype indistinguishable from that of *apoltp* mutants (Figure 3C). LTP RNAi does not reduce the amount of circulating Lpp (Figure 2E); thus, gut lipid accumulation in LTP RNAi animals cannot be caused by reduced Lpp levels. Taken together, these data suggest that LTP must act with Lpp in the gastric caecae and subsets of the anterior and posterior midgut to effect dietary lipid mobilization.

Unlike Lpp and LTP, the vitellogenin-like protein Cv-d is barely detectable in the gut (Figure 2D), and loss of Cv-d does not cause obvious perturbations in gut lipid export (Figure S4A, S4B). Cv-d RNAi does not prevent the development of fertile flies, and Cv-d is not enriched in embryos (Figure S1C), suggesting that unlike canonical vitellogenins it also does not function in embryonic lipid metabolism. This is consistent with the finding that unrelated yolk proteins constitute the major storage proteins in the *Drosophila* embryo [42].

### LTP Loads Lpp with Lipids at the Gut

**Figure 4:**
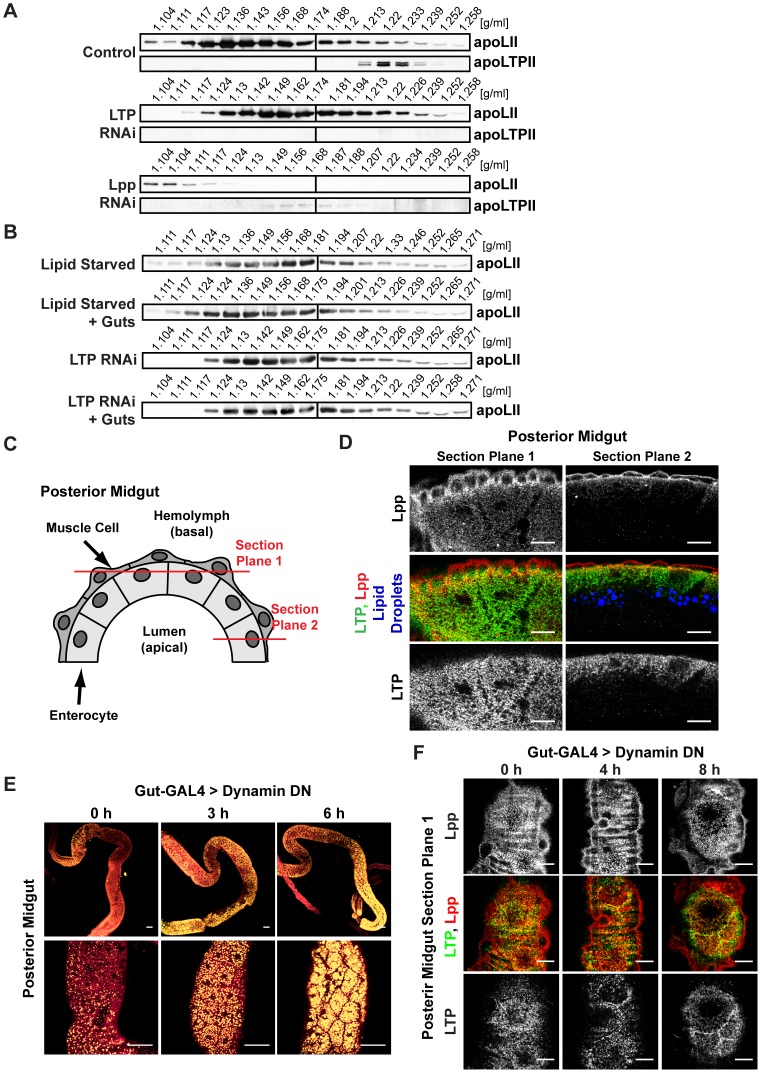
LTP promotes the export of lipids from the gut to Lpp. (A) Immunoblot of isopycnic KBr gradients from LTP or Lpp RNAi hemolymph. Knock-down of LTP increases Lpp density. Knock-down of Lpp decreases LTP density. (B) Immunoblot of isopycnic KBr gradients from gut lipid transfer experiments, showing that LTP facilitates lipid export from the gut to Lpp. (C) Cartoon of the posterior midgut. Optical section planes of (D), (F) are indicated in red. (D) Immunofluorescence of a second larval instar posterior midgut, showing the subcellular localization of Lpp, LTP and neutral lipid droplets. Scale bars = 10 µm. (E) Lipid droplets in the gut of second instar larvae at different time points after the induction of dominant negative (DN) dynamin (shibire), visualized with Nile red. Within a few hours after induction, neutral lipid droplets accumulate to a similar extent as in the gut of lipoprotein-deficient larvae. Yellow: neutral lipids; red: phospholipids. Scale bars = 50 µm. (F) Immunofluorescence of second larval instar posterior midguts at different time points after the induction of dominant negative dynamin, stained for Lpp and LTP. Scale bars = 10 µm.

We were surprised that LTP knock-down produced such a dramatic lipid accumulation phenotype in the gut, because LTP carries a minor proportion of hemolymph lipids. We therefore wondered whether LTP acted catalytically to promote lipid export from the gut to Lpp. To ask whether loss of LTP might change the lipid composition of circulating Lpp, we compared Lpp from wild-type and LTP RNAi hemolymph by density gradient centrifugation. Indeed, LTP RNAi increases Lpp density (Figure 4A), suggesting that Lpp particles contain less lipid when LTP is absent.

To directly test the idea that gut lipids are loaded onto Lpp by LTP, we incubated explanted guts with different combinations of lipoproteins. We collected hemolymph either from LTP RNAi animals or from lipid-starved animals. Both treatments reduce Lpp lipid content (see Figure S9A). However, the hemolymph from lipid-starved animals contains LTP. We incubated the different hemolymph preparations with guts dissected from LTP RNAi animals. After 4 h, we recovered the hemolymph and analyzed Lpp density. In the presence of LTP, Lpp shifts to lower densities after incubation with explanted guts (Figure 4B). In contrast, Lpp density does not change when guts are incubated with LTP-free hemolymph. We conclude that LTP facilitates lipid export from the gut to Lpp. As the LTPs of other insects were shown to have similar lipid transfer activity [26], and also resemble *Drosophila* LTP in density and in the size of two apolipoprotein subunits [35], [36], we suspect that these LTPs are scaffolded by orthologous apoB-family proteins.

*Manduca* LTP can facilitate lipid exchange between Lpp and the fat body. Stimulating adult fat bodies with adipokinetic hormone yields net transfer from the fat body to Lpp [28]. In contrast, in feeding *Manduca* larvae, net lipid flux is from Lpp to the fat body [27]. To ask whether LTP from *Drosophila* larvae promoted loading of Lpp with fat body lipids, we incubated hemolymph containing lipid-poor Lpp derived from LTP RNAi animals with either wild-type fat bodies or LTP RNAi fat bodies. In contrast to the gut, we did not observe net lipid transfer from fat body to Lpp, regardless of the presence of LTP (Figure S5A). Thus, LTP does not appear to promote the loading of Lpp with neutral lipids at the fat body in feeding larvae, similar to the situation in *Manduca*.

Previous work demonstrated that *Manduca* LTP could function as a carrier that shuttles lipids between donor and acceptor lipoproteins [43]. This led us to wonder whether *Drosophila* LTP acted as an intermediate in the transfer of lipids from the gut to Lpp. If so, then loss of Lpp might trap these lipids in LTP particles. To test this, we asked whether removing Lpp altered LTP density. Indeed, LTP shifts to lower density fractions in hemolymph from Lpp RNAi animals (Figure 4A, Figure S5B), suggesting that lipids are loaded onto LTP before being transferred to Lpp.

While MTP transfers lipids to apoB in the secretory pathway of producing cells [2], [3], LTP must function differently in the gut, since both Lpp and LTP are recruited there from circulation. To investigate the LTP/Lpp lipid transfer mechanism, we examined their subcellular localization in the lipid droplet-rich regions of the posterior midgut. Lpp accumulates both in the overlying muscle layer that surrounds the gut, and on the basal (outward facing) side of absorptive enterocytes (Figure 4C, 4D). In contrast, LTP is not detectable in muscle but accumulates strongly in basal regions of enterocytes. Its subcellular localization extends apically to abut the level where lipid droplets are found. There is little obvious subcellular colocalization of LTP and Lpp in the midgut.

The subcellular localization of LTP raised the possibility that it was internalized by enterocytes; we therefore wondered whether endocytosis was required for lipid mobilization from the gut. To address this, we induced expression of dominant negative dynamin in enterocytes and monitored neutral lipid droplets at different times following induction. Within 3 h, neutral lipid droplets accumulate over a broader region of the posterior midgut, and their number within individual enterocytes increases (Figure 4E). By 6 h, most of the posterior midgut is filled with large lipid droplets, similar to the gut of lipoprotein-deficient larvae. Within the same time frame, LTP shifts its subcellular localization to accumulate at the cell boundaries of enterocytes (Figure 4F). In contrast, the subcellular distribution of Lpp appears unaltered. This suggests that endocytosis of LTP may be required for lipid mobilization from the gut. A model consistent with these data is that LTP is internalized by enterocytes, loaded with lipids in an endocytic compartment, and subsequently transfers its lipid cargo to Lpp.

We note that 24 h after induction of dominant negative dynamin, lipid droplets in the gut are strongly reduced (). Since dynamin blocks not only endocytosis, but also some plasma membrane delivery routes, we suspect that blocking endocytosis for longer periods of time might compromise delivery of proteins involved in lipid uptake.

### The Gut Uses LTP to Load Lpp with Medium-Chain DAG and Sterols

**Figure 5:**
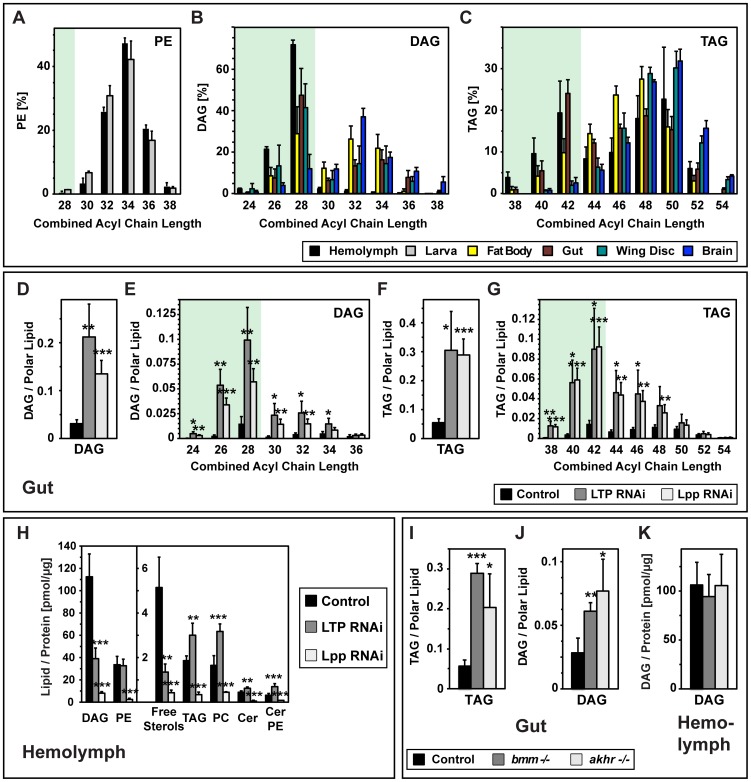
The gut is the major source of Lpp medium-chain diacylglycerol. (A–C) Chain length distribution of fatty acid residues in (A) PE, (B) DAG and (C) TAG in larval hemolymph and tissues, quantified by mass spectrometry (hemolymph n = 8, tissues n = 5). Lipid species are represented as % of their lipid class. See also Figure S6. (D–G) Changes in gut neutral lipids upon LTP or Lpp RNAi, quantified by mass spectrometry (n = 5). (D) total DAG; (E) total TAG; (F) DAG species; (G) TAG species. Acylglycerols are normalized to polar lipid. Knock-down of either LTP or Lpp causes strong accumulation of DAG and TAG in the gut, in particular of those species containing medium-chain fatty acid residues. (H) Changes in hemolymph lipids upon LTP or Lpp RNAi, quantified by mass spectrometry (control n = 8; LTP n = 6; Lpp RNAi n = 7; free sterols n = 4). Lipids are normalized to hemolymph protein. LTP knock-down specifically decreases DAG and sterols. Lpp knock-down strongly decreases all lipids. (I–J) Neutral lipid changes in *bmm* and *akhr* mutant larvae, quantified by mass spectrometry (n = 3). (I) gut DAG; (J) gut TAG, normalized to polar lipid. (K) hemolymph DAG, normalized to hemolymph protein. TAG and DAG levels in the gut increase in mutant larvae, but Lpp DAG levels are not perturbed.

The vast majority of lipids in the hemolymph are carried by Lpp, and Lpp RNAi reduces the amount of all hemolymph lipid species over 10-fold (Figure 5H). To ask which lipids depended LTP for their transfer to Lpp, we quantified changes in hemolymph lipids of LTP RNAi animals. LTP RNAi specifically reduces the levels of medium-chain DAG (DAG 26, DAG 28) and sterols (Figure 5H, Figure S5D) by about 70%. In contrast, levels of PE, the major polar Lpp lipid, are not changed. The amounts of several minor Lpp lipid classes (PC, TAG, sphingolipids) increase slightly. These data suggest that LTP specifically facilitates loading of DAG and sterols onto Lpp.

We reasoned that cargo transferred to Lpp by LTP might specifically accumulate in the gut upon either LTP or Lpp knock-down. We therefore asked whether DAG and sterol increased under these conditions. Wild-type guts contain both the medium-chain DAG found in Lpp and smaller amounts of long-chain DAG (Figure 5B, Figure S6) whose combined acyl chain length resembles those of cellular and Lpp phospholipids (Figure 5A). Upon LTP or Lpp RNAi, medium-chain DAG increases 5–8 fold with respect to polar lipids (Figure 5D, 5E). Long-chain DAG increases moderately, and contributes less to the total elevation in gut DAG. These data confirm that the gut uses LTP to export medium-chain DAG to Lpp. Although the gut must also be the source of Lpp sterol (*Drosophila* are sterol auxotrophs), sterols do not accumulate in this organ upon lipoprotein knock-down (Figure S7A, S7B). It is possible that sterol esterification may increase when export is blocked; however our current methods do not allow us to detect sterol esters.

Strikingly, loss of either LTP or Lpp also causes a strong increase in TAG in the gut (Figure 5F). Since the minor amounts of TAG normally present Lpp particles do not decrease upon LTP RNAi (Figure 5H), this cannot reflect a block in TAG export to Lpp. Interestingly, medium-chain TAG is most strongly elevated (Figure 5G). This suggests that some medium-chain fatty acids eventually exported as DAG can be stored as TAG, when export from the gut is blocked.

We wondered whether incorporation of medium-chain fatty acids into TAG was an obligate intermediate in the production of medium-chain DAG. To address this, we perturbed the two well-characterized lipolytic systems known to be required for hydrolysis of TAG in the *Drosophila* fat body – the Adipocyte Triglyceride Lipase homologue, Brummer (Bmm), and the Adipokinetic Hormone Receptor (AKHR) regulated lipase system [13], [44]. We first quantified gut TAG and DAG species in *bmm* and *akhr* mutant larvae. Loss of either Bmm or AKHR causes TAG accumulation that is biased towards medium-chain species – similar to the effect of lipoprotein knock-down (Figure 5I, Figure S8A, S8B). Thus, the gut requires both lipolytic systems to mobilize TAG at a normal rate. Despite this, neither perturbation reduces medium-chain DAG in the hemolymph (Figure 5K, Figure S8C). Levels of medium-chain DAG in the gut actually increase slightly (Figure 5J, Figure S8D). We conclude that even under these conditions where TAG storage is favored over lipolysis, the gut can supply normal levels of DAG to Lpp.

To ask whether these lipolytic pathways might function redundantly to generate Lpp DAG, we knocked down Bmm and AKHR in enterocytes, alone and in combination, and measured resulting changes in hemolymph DAG. While neither knock-down alone reduces hemolymph DAG (similar to the effect of single mutants) simultaneous knock-down lowers hemolymph DAG by 15% (Figure S8E). These data suggest that some Lpp medium-chain DAG is derived from medium-chain TAG by Bmm and AKHR-dependent lipolysis. However, other mechanisms suffice to generate the majority of Lpp medium-chain DAG.

### Lpp Medium-Chain DAG Is Derived Both from Dietary Lipids and *De Novo* Synthesis in the Gut

We were intrigued by the distinctive fatty acid composition of Lpp DAG. The combined acyl chain length in these DAG species (26–28 carbons) differs not only from that of phospholipids, but also from that of dietary lipids – both contain almost exclusively long-chain fatty acids (32–36 carbons) (Figure 1G, Figure 5A; M. Carvalho et al., submitted). These observations raise questions about the source of the medium-chain fatty acids in Lpp DAG. One possibility is that they are derived from dietary fatty acids by processing mechanisms such as chain length shortening. Alternatively, they may be generated *de novo* from non-lipid dietary components such as sugars.

To determine the contribution of dietary lipids to Lpp medium-chain DAG, we compared levels of hemolymph DAG in lipid-fed and lipid-starved animals. Lipid starvation increases the density of hemolymph Lpp (Figure S9A). Thus, lipid-starved animals produce similar amounts of Lpp, but these particles contain less lipid. Furthermore, lipid starvation reduces the ratio of medium-chain DAG to polar lipids in Lpp by about 2-fold (Figure S9B). However, lipid starvation affects Lpp density and DAG content more mildly than LTP RNAi, which reduces the ratio of DAG to polar lipid by about 3-fold. This raises the possibility that only part of the medium-chain DAG loaded onto Lpp by LTP is derived from dietary lipids.

**Figure 6:**
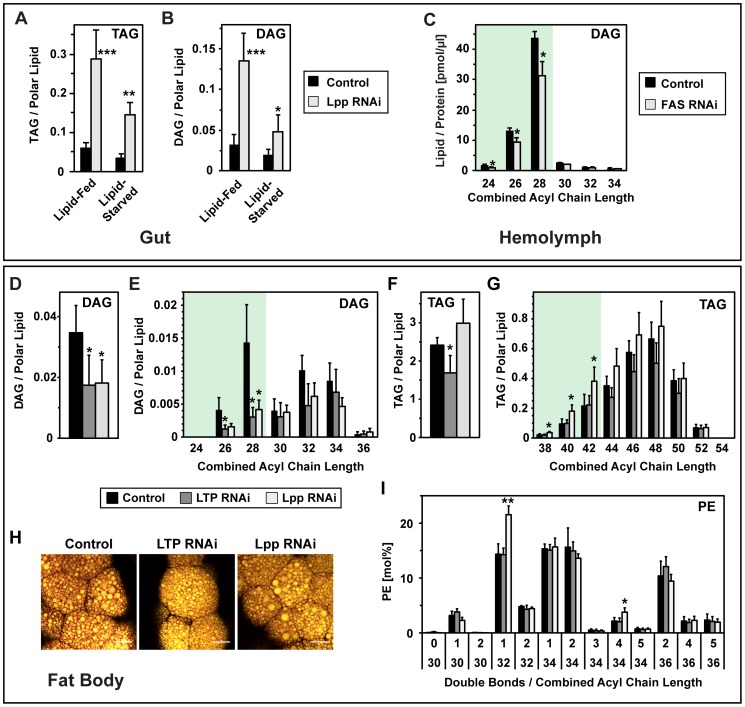
Lipid synthesis capacity of gut and fat body. (A), (B) Changes in gut (A) TAG and (B) DAG of lipid-fed and lipid-starved larvae upon Lpp RNAi, quantified by mass spectrometry (n = 3). Lpp knock-down causes neutral lipid accumulation in the gut, even when lipids are not provided with the diet. (C) Changes in hemolymph DAG upon gut-specific FAS RNAi, quantified by mass spectrometry (n = 3). DAG is normalized to hemolymph protein. FAS knock-down causes a decrease in hemolymph DAG. (D–G) Changes in fat body neutral lipids upon LTP or Lpp RNAi, quantified by mass spectrometry (n = 5). (D) total DAG; (E) DAG species; (F) total TAG; (G) TAG species. Knock-down of either lipoprotein decreases medium-chain DAG. (H) Larval fat body lipid droplets upon LTP or Lpp RNAi, visualized with Nile red. Lipoprotein knock-down does not reduce lipid droplet levels. Yellow; neutral lipid; red: phospholipid. Scale bars = 20 µm. (I) Changes in fat body PE species upon LTP or Lpp RNAi, quantified by mass spectrometry (n = 5). Acylglycerols are normalized to polar lipid. PE is represented as mol% of polar lipids.

To explore the contribution of endogenous synthesis, we asked to what extent neutral lipid accumulation in the gut of Lpp RNAi larvae depended on dietary lipids. We knocked down Lpp in lipid-fed and lipid-starved larvae, and quantified neutral lipids in the gut. Although lipid starvation slightly reduces the amount of TAG and DAG in wild-type guts, these lipids accumulate dramatically when Lpp levels are reduced - both in lipid-fed and in lipid-starved larvae (Figure 6A, 6B). On both diets, neutral lipid species containing medium-chain fatty acids increase most strongly in response to Lpp RNAi (Figure S9C). This supports the idea that part of the medium-chain DAG present in Lpp derives from endogenous synthesis in the gut.

*Drosophila* Fatty Acid Synthase (FAS) can synthesize medium-chain fatty acids [45], raising the possibility that it generates fatty acids for the medium-chain DAG present in Lpp. To investigate the contribution of fatty acid synthesis in the gut to Lpp DAG, we knocked down FAS in this organ, and quantified hemolymph DAG. FAS RNAi decreases Lpp DAG by 30% (Figure 6C). Therefore, even when fatty acids are supplied by the diet, a significant proportion of Lpp DAG contains fatty acids derived from endogenous synthesis in the gut.

### The Fat Body Autonomously Maintains Its TAG Stores within a Narrow Range

The insect fat body is a major site of lipid synthesis, storage and export [25]. How does the balance of lipid import and export affect the lipid composition of this organ? We first examined the contribution of lipid delivery from the gut by blocking this transport route through RNAi-mediated LTP knock-down. Fat bodies of LTP RNAi animals contain much less sterol than those of wild-type, consistent with the sterol auxotrophy of *Drosophila* (Figure S7A, S7C). Interestingly, LTP RNAi fat bodies also contain much less medium-chain DAG (Figure 6D, 6E). Thus, the fat body does not maintain medium-chain DAG levels when export from the gut is blocked.

To what extent is delivery of lipids from the gut required to build TAG stores in the fat body? The wild-type fat body contains large amounts of TAG with predominantly long-chain fatty acids – unlike the gut, which contains similar amounts of medium-chain and long-chain TAG (Figure 5C, Figure S6). Removing lipids from the diet does not reduce the amount of TAG stored in the fat body (Figure S9D). Thus, endogenous synthesis of fatty acids from non-lipid sources suffices to build TAG stores in the fat body. Impairing lipid delivery from the gut by LTP RNAi reduces fat body TAG storage by 30% (Figure 6F, 6G) but does not obviously alter the morphology of neutral lipid droplets (Figure 6H). Interestingly, blocking both Lpp-dependent lipid import and export from the fat body using Lpp RNAi does not lower fat body TAG levels at all. Taken together, these data suggest that homeostatic mechanisms in the fat body, presumably involving endogenous lipid synthesis, can compensate for reduced lipid delivery from the gut.

The fat body produces Lpp particles rich in long-chain PE species with a combined acyl chain length of 32 or 34 carbons, and 1 double bond (PE 32∶1 and 34∶1; Figure S10A), which are also major phospholipid species in fat body membranes. We wondered how impaired Lpp production might affect the levels of these PE species. Loss of Lpp, but not LTP, increases the level of PE 32∶1 in the fat body about 1.5-fold (Figure 6I). However, this is modest compared with the 5-fold increase in DAG in the gut that occurs upon lipoprotein knock-down. Since the gut expands its stores of medium-chain TAG when export of DAG is blocked, we considered the possibility that the fat body might increase its stores of long-chain TAG when PE export is blocked. However, this is not the case; Lpp RNAi does not significantly increase the amount of long-chain TAG in the fat body (Figure 6G). These data indicate that homeostatic mechanisms in the fat body maintain TAG storage in a narrow range.

### Neutral Lipid Storage in the Wing Imaginal Disc Depends on Lipoprotein-Mediated Lipid Delivery

**Figure 7:**
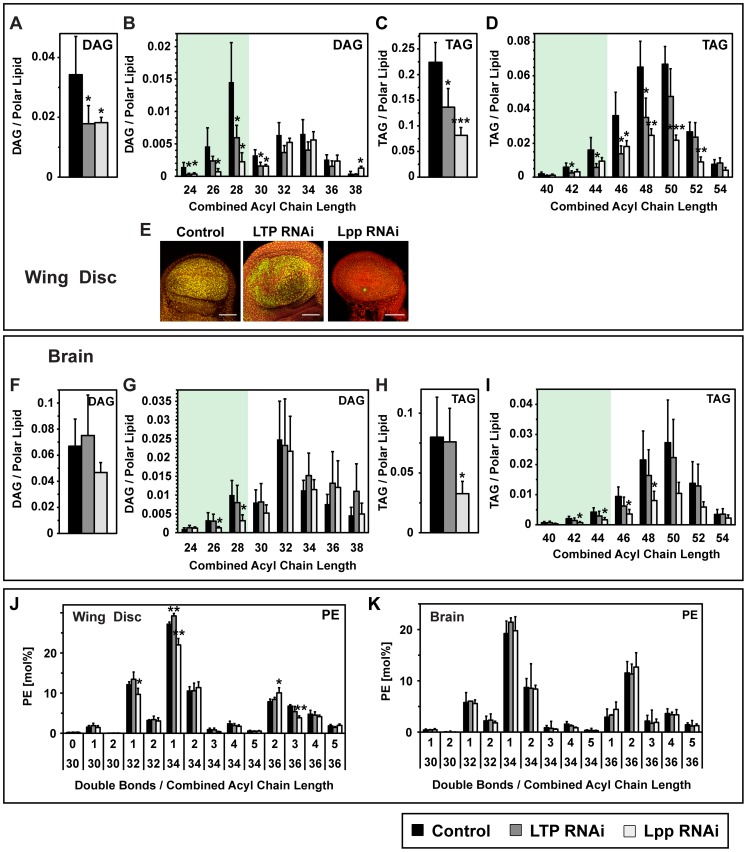
Lpp supplies lipids to peripheral organs. (A–D) Changes in wing disc neutral lipids upon LTP or Lpp RNAi, quantified by mass spectrometry (n = 5). (A) total DAG; (B) DAG species; (C) total TAG; (D) TAG species. Knock-down of either lipoprotein decreases medium-chain DAG and TAG species. Lpp knock-down in addition decreases long-chain TAG. (E) Lipid droplets of wing discs upon LTP or Lpp RNAi, visualized with Nile red. Lpp knock-down decreases wing disc lipid droplets more strongly than LTP knock-down. Yellow: neutral lipids; red: phospholipids. Scale bars = 50 µm. (F–I) Changes in brain neutral lipids upon LTP or Lpp RNAi, quantified by mass spectrometry (n = 5). (F) total DAG; (G) DAG species; (H) total TAG; (I) TAG species. Lpp knock-down decreases DAG and TAG, whereas LTP knock-down does not affect brain neutral lipids. (J),(K) Changes in PE species in (J) wing disc and (K) brain, quantified by mass spectrometry (n = 5). The major Lpp PE species (PE 32∶1, PE34∶1) decrease upon Lpp knock-down in the wing disc, but not in the brain. Acylglycerols are normalized to polar lipid. PE is represented as mol% of polar lipids.

Our previous work showed that Lpp is required for neutral lipid storage in the wing imaginal disc [29]. We wondered whether LTP-dependent mobilization of gut lipids onto Lpp contributed to the TAG and DAG stores of the wing disc. To address this, we quantified different species of neutral lipids in wild-type, Lpp RNAi and LTP RNAi wing discs. Wild-type wing disc cells contain medium-chain DAG, like that in Lpp, and also long-chain DAG (Figure 5B, Figure S6). Loss of either Lpp or LTP specifically depletes medium-chain DAG without affecting long-chain DAG (Figure 7A, 7B). This suggests that a large fraction of the medium-chain DAG in wing disc cells is derived from the gut via LTP and Lpp.

Total TAG levels in wing disc cells are reduced 60% by Lpp RNAi and 40% by LTP RNAi (Figure 7C). Consistent with this, Lpp RNAi strongly reduces the size and number of neutral lipid droplets in the wing disc, while LTP RNAi has more modest effects (Figure 7E). These data confirm that Lpp-mediated lipid delivery is needed for wing disc cells to store normal levels of TAG. They further indicate that mobilization of DAG from the gut via LTP and Lpp contributes to wing disc TAG stores. However, other Lpp lipids partially support wing disc TAG storage, at least when gut lipid mobilization is blocked. Medium-chain TAG species are reduced equally by Lpp and LTP RNAi, whereas the effects of LTP RNAi on long-chain TAG species is weaker (Figure 7D). This raises the possibility that Lpp long-chain PE, which does not decrease upon LTP RNAi, contributes fatty acids to long-chain TAG stored in wing disc cells.

### TAG Storage in the Brain Depends on Lpp but Does Not Require Gut Lipid Mobilization by LTP

The *Drosophila* brain is shielded from the circulation by a blood-brain barrier similar to that of mammals. Nevertheless, Lpp crosses this barrier [46], raising the possibility that cells in the brain may derive lipids from the circulation. To investigate this, we analyzed lipid profiles in the brains of wild-type, LTP RNAi and Lpp RNAi animals.

Lpp RNAi decreases medium-chain DAG, but not long-chain DAG in the brain (Figure 7F, 7G), and reduces total brain TAG by 60% (Figure 7H, 7I). In contrast, LTP RNAi does not affect neutral lipids in brain cells significantly. Thus, normal TAG storage in the brain requires Lpp-mediated lipid delivery, but it is not limited by the LTP-dependent loading of Lpp with DAG in the gut.

### Phospholipid Composition of the Wing Disc and Gut Is Influenced by Lpp-Mediated Lipid Delivery

To what extent do different tissues synthesize their own membrane lipids? Do they also rely on Lpp to deliver some membrane lipids? Because *Drosophila* cannot synthesize sterols, it is unsurprising that sterol accumulation in peripheral tissues depends on Lpp and LTP (Figure S7A) [18]. To investigate whether Lpp was important for delivery of other membrane lipids, we quantified the polar lipid composition of tissues from LTP and Lpp RNAi animals. Wing disc and gut from Lpp RNAi animals contain about 20% less PE 32∶1 and PE 34∶1 than those of wild-type (Figure 7J, Figure S10B). Interestingly, these PE species are not only major membrane constituents, but are also precisely those species that are most abundant in Lpp (Figure S10A). In contrast, we did not observed a reduction in any species of PC, which is abundant in membranes, but only a minor component of Lpp (Figure S10C). These observations suggest that wing disc and gut cells cannot completely compensate for the loss of Lpp-derived PE species by increasing endogenous PE synthesis. They further raise the possibility that Lpp PE species might be directly incorporated into cell membranes without remodeling.

In contrast to wing disc and gut, the brain can maintain normal levels of all phospholipids including PE 32∶1 and PE 34∶1 even when Lpp levels are strongly reduced by RNAi (Figure 7K, Figure S10C, S10D). Thus, while the brain requires Lpp delivery to store normal levels of TAG, its phospholipid composition is autonomously controlled.

## Discussion

**Figure 8:**
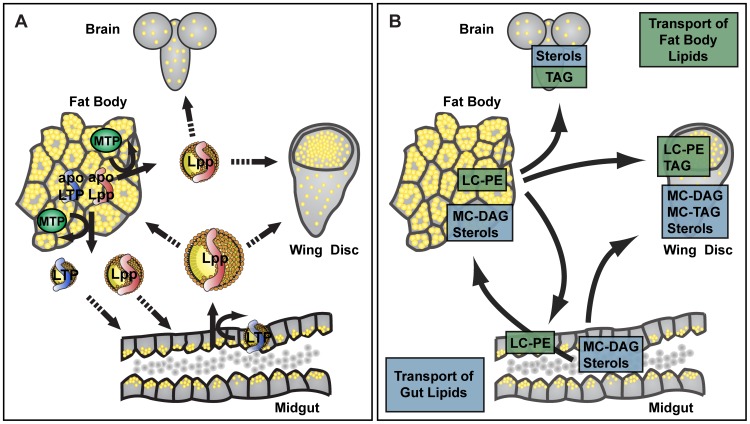
*Drosophila* lipoprotein metabolism. (A) *Drosophila* lipoproteins and lipid transfer proteins. The fat body assembles the apoB-family lipoproteins Lpp and LTP in an MTP-dependent process. Lpp and LTP are subsequently recruited to the gut, where LTP promotes loading of further lipid onto Lpp. As the major hemolymph lipid carrier, Lpp transports lipids from fat body and gut to other tissues. (B) Model for major inter-organ lipid transfer routes of feeding larvae, deduced from lipoprotein RNAi phenotypes. The fat body produces Lpp particles rich in long-chain PE. The gut mainly exports sterols and medium-chain DAG. Sterols are delivered to all organs. Medium-chain DAG exported from the gut is delivered to fat body and wing disc, and contributes to normal TAG storage. Independently of this route, Lpp lipids can also be delivered to the wing disc and brain, to support TAG stores. Finally, Lpp lipids contribute to the PE composition of wing disc and gut membranes. Note that loss of function experiments reveal only those lipid transport routes that cannot be compensated by other mechanisms.

How disturbed lipoprotein metabolism affects lipid composition in individual organs is insufficiently understood. *Drosophila* could provide a useful model in which to study this problem, but its lipoproteins had not been well characterized. Here, we outline the basic features of the lipoprotein metabolism of *Drosophila* larvae, and its relevance for tissue-specific fat storage and membrane lipid composition (Figure 8).

The major inter-organ lipid transport routes in *Drosophila* are executed by a single lipoprotein – Lpp, which is scaffolded by the apoB homologue apoLpp. Its major polar lipid constituents are long-chain PE and sterols, and its major neutral lipid is medium-chain DAG. Lpp lipidation takes place in two consecutive steps, which require distinct lipid transfer proteins, MTP and LTP, and take place in different organs – fat body and gut. ApoLpp is translated and lipidated in the fat body by an MTP-dependent mechanism, resulting in the formation of dense Lpp particles rich in PE. These are recruited to the gut, where they are further loaded with DAG and sterols through the activity of LTP. Thus, although Lpp originates in the fat body, it is loaded both with fat body and gut lipids.

Lipidation of mammalian apoB, like that of *Drosophila* apoLpp, proceeds in two distinct steps, formation of primordial phospholipid-rich lipoprotein particles, and subsequently acquisition of bulk neutral lipid [2]. However, this process occurs entirely in the secretory pathway of producing cells. MTP has been proposed to be required both for initial transfer of phospholipids, and for the recruitment of TAG to the ER lumen for incorporation into lipoproteins [47], [48], [49], [50]. Interestingly, *Drosophila* MTP has been shown to promote the secretion of apoB-containing lipoproteins from COS cells, and to transfer phospholipids, but not TAG, between liposomes [31], [51]. This suggested that MTP acquired the ability to transfer TAG in the vertebrate lineage. Our experiments show that *Drosophila* MTP is required for the production of the two *Drosophila* apoB-family lipoproteins Lpp and LTP *in vivo*; they further show that MTP is insufficient to load Lpp with normal quantities of DAG, the major neutral lipid of Lpp. These data support the idea that MTP originally evolved to promote the assembly of phospholipid-rich apoB-family lipoproteins.

The novel *Drosophila* apoB-family lipoprotein LTP shares many properties with the Lipid Transfer Particle purified from the hemolymph of several insects, including *Manduca* and *Locusta* [35], [36]. The scaffolding proteins of *Drosophila* LTP, apoLTPI and apoLTPII, are generated from a single precursor, apoLTP. Orthologous apoB-family proteins of other insects are therefore plausible candidates for the scaffolding proteins of their LTP particles. Insect LTPs were shown to contain a third, small protein subunit, apoLTPIII [36], [52]. Our biochemical experiments do not address whether *Drosophila* LTP might contain an apoLTPIII subunit, because LTP is of such low abundance that silver staining barely detects the much larger apoLTPI. Sequence analysis of apoLTP does not suggest the existence of a protease cleavage site that could give rise to a protein of the size of apoLTPIII, and neither apoLTPI nor apoLTPII antibodies detect an additional protein of this size. Thus, if apoLTPIII exists in *Drosophila*, it is not likely to be derived from the apoLTP precursor.

The function of LTP as a lipid transfer protein rather than a carrier of bulk hemolymph lipid uncovers surprising evolutionary plasticity of the apoB lipoprotein family. Insect LTPs have been studied *in vitro* in a wide range of systems [38]. In different contexts, they have been shown to facilitate the exchange of DAG and phospholipids between Lpp and fat body or gut [26], [27], [28], and even between insect and human lipoproteins of different densities [52], [53], [54]. Our studies of feeding *Drosophila* larvae indicate that only a subset of the lipid transfer activities of LTP may be relevant under specific metabolic conditions *in vivo*. LTP moves DAG and sterols from the larval gut onto Lpp. However, it does not facilitate significant net transfer of fat body lipids to Lpp. Consistent with this, radiolabeling experiments showed that the rate of DAG transfer from larval *Manduca* fat body to Lpp exceeds the rate of the reverse process [27]. This may reflect a dominance of nutritional lipid uptake and fat storage in feeding larvae.

Although we have been unable to identify a *Drosophila* HDL-like lipoprotein, we note that LTP and Lpp share some functional features with mammalian HDL, despite being scaffolded by unrelated apolipoproteins. Together, Lpp and LTP mediate efflux of sterols from the gut to circulation. Conceivably, other tissues that recruited both lipoproteins might efflux sterol for reverse transport.

While it is clear that dietary lipids do contribute to Lpp DAG, the gut does not directly incorporate dietary fatty acids into DAG destined for export. The long-chain fatty acids that predominate in the diet strikingly differ from the medium-chain fatty acids in Lpp DAG (M. Carvalho et al., submitted). A possible explanation is that the gut remodels dietary fatty acids, conceivably via limited β-oxidation. Interestingly, the gut is also a lipogenic organ and a significant fraction of the medium-chain fatty acids found in Lpp DAG derives from *de novo* fatty acid synthesis in this organ. In more primitive animals, such as *Caenorhabditis elegans*, lipid uptake, storage and lipogenesis all occur in the gut [55]. More complex animals, including *Drosophila*, have developed separate organ systems for lipid storage and lipogenesis. However, our data show that this separation of functions is not absolute in the fly. Rather, other nutrients such as amino acids or sugars might be partially converted to lipid by the gut, instead of being transported intact into circulation. It would be interesting to ask what circumstances favor this conversion. Intriguingly, *de novo* lipogenesis has been observed in the mammalian gut, especially under conditions of insulin resistance, and has been proposed to contribute to the postprandial dyslipidemia observed in this state [56]. *Drosophila* may be a useful model to explore this problem.

Gut and fat body differ in how they respond to blockage of lipid export to Lpp. Enterocytes vastly and rapidly expand their normally moderate stores of medium-chain DAG and TAG. This occurs even in the absence of dietary lipids, when exported lipids are derived from endogenous fatty acid synthesis. Thus, the gut has a flexible capacity for lipid storage. In contrast, the larval fat body maintains its neutral lipid stores within tight limits. When lipoprotein transport is blocked, endogenous lipid synthesis from other dietary components may suffice to build the large TAG stores of this organ. Furthermore, even though the fat body normally supplies the entire animal with large amounts of lipoproteins, TAG stores hardly increase when Lpp is not produced. Homeostatic mechanisms must maintain fat body TAG levels. In this way, the fat body differs from the gut, which accumulates fat when lipoprotein export is blocked, similar to mammalian gut and liver [5].

Peripheral tissues cannot maintain normal TAG levels in the absence of Lpp. The wing disc depends on Lpp for a large fraction of its fat stores. Interestingly, our work indicates that lipid delivery from the fat body and gut differently contributes to wing disc neutral lipids. TAG species containing medium-chain fatty acids depends on LTP and Lpp-mediated DAG mobilization from the gut. TAG species containing long-chain fatty acids also depend on Lpp-mediated lipid delivery, but are less affected by a blockage of DAG export from the gut. As Lpp is produced in the fat body, this suggests that long-chain TAG in wing discs may be derived from lipids supplied by the fat body. The most abundant source of long-chain fatty acids in Lpp is PE, which raises the possibility that wing discs use Lpp phospholipids to build cellular fat stores. Consistent with this, cultured murine hepatocytes convert a significant fraction of LDL or HDL-derived PC to TAG [57], [58], although the *in vivo* relevance of this pathway remains to be explored. However, Lpp still contains reduced amounts of medium-chain DAG when LTP-mediated lipid loading is impaired. Thus, long-chain fatty acids in wing disc TAG might also derive from elongation of medium-chain fatty acids. Interestingly, although medium-chain DAG is the most abundant lipid transported through circulation, tissues store only minor amounts of neutral lipid containing medium-chain fatty acids. This would be consistent with the idea that tissues either elongate these fatty acids or subject them to β-oxidation.

The brain also requires Lpp-mediated lipid delivery to build its TAG stores. Interestingly, the brain stores normal levels of TAG when gut lipid mobilization is inhibited. While this does not exclude the possibility that the brain may directly acquire lipids from the gut under normal conditions, it indicates that TAG levels in this organ are more resistant to fluctuations in nutritional conditions than those in the wing disc.

In addition to providing fatty acids for neutral lipid storage, lipoproteins also influence the phospholipid composition of wing disc and gut: Lpp knock-down specifically reduces those PE species that are most abundant in Lpp. This suggests that Lpp might directly deliver PE to the cellular membranes of wing disc and gut. It further raises the possibility that phospholipid synthesis in other tissues could have organism-wide effects on membrane lipid composition. Since PE-rich Lpp particles are assembled in the fat body, this tissue is a likely source of these lipids. However, the brain does not depend on Lpp to maintain its normal membrane phospholipid composition. Furthermore, our previous work suggested that the brain is more resistant to sterol depletion than other tissues [59]. In general, these data indicate that the lipid composition of the brain is more tightly and autonomously controlled than that of other tissues.

In mammals, cellular lipid synthesis and lipid supply from circulation are coordinated through the SREBP pathway [60], [61]. Since *Drosophila* SREBP is regulated by PE instead of sterols [62], it will be interesting to explore whether altered PE levels in Lpp-deprived wing discs might activate SREBP signaling and increase lipid synthesis or lipoprotein uptake. If true, coordination of cellular lipid synthesis with lipid supply through lipoproteins is an evolutionarily conserved function of the SREBP pathway.

Lipoproteins transport large amounts of lipids through circulation – including many of the polar and neutral lipid species present in cells. Our data indicate that in *Drosophila*, individual organs utilize lipoprotein-derived lipids not only for fat storage but also for membrane homeostasis. ApoB-deficient human patients, and patients with dyslipidemia suffer from various abnormalities in peripheral tissues. Our data suggest that it may be worthwhile to examine how these perturbations alter the membrane lipid composition of affected tissues.

## Materials and Methods

### Fly Techniques

Flies were raised on food containing yeast, yeast extract, soy peptone, sucrose, and fructose. For lipid starvation experiments, larvae were fed lipid-depleted food, supplemented with sterols [59]. Unless otherwise stated, feeding late third instar larvae were used for experiments.

### Antibodies

ApoLII and apoLI antibodies were described previously [20], [29]. Mouse anti-α-tubulin was provided by Sigma.

ApoLTPII, apoLTPI and MTP antibodies were raised in rabbit, Cv-d antibodies in guinea pig.

### RNAi

Inducible RNAi lines were crossed with lines harboring heat-shock-inducible flippase and the GAL4 driver. RNAi was initiated by heat shock during early larval development. Controls are stage-matched larvae from the same cross lacking the GAL4 driver. RNAi was driven with Adh-GAL4, which is mostly active in the fat body. In experiments addressing the contribution of the fat body to hemolymph lipoprotein levels, RNAi was driven with Lpp-GAL4, which is exclusively active in the fat body during larval stages. RNAi driven with either Adh-GAL4 or Lpp-GAL4 reduced hemolymph Lpp and LTP to the same extent.

### Hemolymph Isolation and Lipoprotein Fractionation

Larvae were bled in PBS. Hemocytes and cell fragments were removed by centrifugation for 30 min at 1500 g and subsequently for 30 min at 16 000 g. Note that stronger centrifugation pellets a large fraction of hemolymph lipoproteins. Isopycnic centrifugation in KBr gradients was essentially performed as described [63].

### Tissue Staining

Immunostaining and Nile red staining of tissues was performed as described [20], [29]. For co-staining with antibodies, lipid droplets were visualized with BODIPY 493/503 (Invitrogen).

### *Ex Vivo* Lipid Transfer Experiments with Explanted Guts

Explanted guts from early 3rd instar LTP RNAi larvae were incubated for 4 h at 25°C with hemolymph prepared from LTP RNAi larvae or lipid-starved larvae, diluted in Grace's insect medium. Subsequently, lipoprotein density was determined by isopycnic centrifugation and immunoblotting.

### Induction of Dominant Negative Dynamin

A dominant negative allele of dynamin (shibire K44A) was expressed in enterocytes in a time-controlled manner with MyoIA-GAL4, Tubulin-GAL80^TS^.

### Shotgun Lipidomics Mass Spectrometry

Lipids from hemolymph and tissue homogenates were extracted and analyzed by shotgun mass spectrometry in positive ion mode as described in [59]. Sterols were quantified according to [64]. For supporting mass spectrometry data, see Figure S11.

For more detailed protocols, fly strains, and the generation of *mtp* and *apoltp* mutants, RNAi transgenes and antibodies see Text S1.

## Supporting Information

Figure S1 *Drosophila* lipoproteins and their lipid content. (A) Specificity of apolipoprotein antibodies. An immunoblot of hemolymph from feeding third instar larvae was consecutively probed for the individual proteins. The apoLTPII antibody recognizes two proteins of similar molecular weight. ApoLTP harbors two closely spaced furin consensus sequences (see Figure 1A), indicating that the two forms of apoLTPII are generated by alternative use of either cleavage site. Note that MTP likewise exists in two isoforms of similar size (see Figure 2D, 2E); however, we could not identify putative protease consensus sites in the MTP sequence. (B) Coomassie staining of hemolymph proteins fractionated on an isopycnic Optiprep gradient. Lpp is the only detectable protein present in the lower-density fractions 1–9. Note that NuPAGE MES buffer was used for electrophoresis of this gradient, whereas electrophoresis of the gradients shown in Figure 1C, 1D was performed with Tris-glycine buffer. (C) Immunoblot showing that apoLpp, apoLTP, Cv-d and MTP proteins are present in embryos, third instar larvae and adult flies. (D) Distribution of lipid classes in the hemolymph density gradient from Figure 1E. Shown is the % lipid of each lipid class present in each fraction. (E) Ceramide-Phosphorylethanolamine (CerPE) present in different fractions of the hemolymph density gradient of Figure 1E. Shown is the total amount of CerPE present in each gradient fraction from 1 µl hemolymph. Note that CerPE 2∶2 and 2∶3 (2 double bonds, 2 or 3 hydroxyl groups) partially co-fractionate with LTP, whereas CerPE 1∶2 (1 double bond, 2 hydroxyl groups) is confined to the Lpp fractions.
(TIF)

Figure S2 Apolipoprotein transcripts are not detectable in the gut. Reverse transcription PCR showing that apolipoprotein transcripts can be detected in the fat body, but not in the gut of third instar larvae. Primer pairs were designed to span small introns to preclude contamination with genomic DNA. Note that actin transcripts can be readily detected in cDNA preparations of both fat body and gut.
(TIF)

Figure S3 Phenotypes of *apolpp*, *apoltp* and *mtp* mutants. (A) Schematic representation of the *mtp* null allele *mtp^Δex1^*. (B) Immunoblot of larvae homozygous for *mtp^Δex1^*. Mutant animals lack any detectable MTP protein. (C) Wild-type and *mtp^Δex1^* larvae 4 days after egg laying. Wild-type animals have reached the third larval instar. Animals homozygous for *mtp^Δex1^* arrest in the first larval instar. (D) Immunofluorescence of the posterior midgut from first instar *mtp^Δex1^* mutant larvae showing that *mtp* mutant guts lack detectable Lpp and LTP. Basolateral membranes are marked with discs large (Dlg). Scale bars = 20 µm. (E) Immunblot of first instar *mtp^Δex1^* larvae in which lipoprotein production was rescued by fat body-specific expression of MTP with Lpp-GAL4. ApoLpp cleavage is impaired in *mtp^Δex1^* larvae, but restored through fat body-specific expression of MTP. (F) Fat body-specific expression of MTP with Lpp-GAL4 in *mtp^Δex1^* larvae rescues intestinal lipid mobilization. Lipid droplets of first instar posterior midguts are visualized with Nile red. Yellow: neutral lipids, red: phospholipids. Scale bars = 20 µm. (G) Immunofluorescence showing that Lpp is produced in yolk cells of stage 14 embryos, but then spreads throughout the whole embryo. Lpp expression is visualized with Lpp-GAL4-driven membrane GFP (CD8-GFP). Nuclei are visualized with DAPI. Scale bars = 50 µm. (H) Knock-down efficiency of Lpp and LTP in third instar larvae, 4 days after induction of RNAi. ApoLI and apoLTPI levels in whole larval extracts were quantified by immunoblotting. Note that Lpp RNAi entails a concomitant partial reduction of LTP. Error bars indicate ±SD (n = 5). (I) Schematic representation of the *apoltp* alleles *apoltp^Δex1A^* and *apoltp^Δex1B^*. (J) Immunoblot of second instar larvae homozygous for *apoltp^Δex1A^* and their hemolymph. Mutant animals show strongly reduced apoLTPI and apoLTPII levels, with apoLII and MTP being unaffected. (K) Immunoblot of first instar larvae homozygous for *apoltp^Δex1B^*. Mutant animals show strongly reduced apoLTPII levels, with apoLII and MTP being unaffected. (L) Wild-type, *apoltp^Δex1A^* and *apoltp^Δex1B^* larvae 4 days after egg laying. Wild-type animals have reached the third larval instar. Animals homozygous for *apoltp^Δex1A^* arrest in the second larval instar, animals homozygous for *apoltp^Δex1B^* arrest in the first larval instar. (M) Intestinal lipid droplets of second instar *apoltp^Δex1A^* mutant larvae visualized with Nile red. Yellow: neutral lipids; red: phospholipids. Mutant larvae strongly accumulate lipid droplets in the anterior midgut (not shown) and posterior midgut. Scale bars = 50 µm. Moderate lipid accumulation also occurs in the gastric caecae. Scale bars = 20 µm.
(TIF)

Figure S4 Phenotypes of RNAi against the vitellogenin-like protein Cv-d. (A) Lipid droplets in the posterior midgut, fat body and wing disc of Cv-d RNAi third instar larvae visualized with Nile red. Yellow: neutral lipids; red: phospholipids. Cv-d knock-down does not obviously perturb lipid droplets in any organ. Scale bars = 50 µm. (B) Unesterified sterols of the gut, fat body and wing disc of Cv-d RNAi third instar larvae visualized with Filipin. Sterols can still be detected in all organs upon Cv-d knock-down. Scale bars = 50 µm.
(TIF)

Figure S5 Lipid mobilization from fat body and gut. (A) Immunoblot of isopycnic KBr gradients from lipid transfer experiments between fat bodies and LTP RNAi hemolymph containing HA-Lpp. Lpp density does not decrease upon incubation with fat bodies, regardless the presence of LTP. Note that the density of fractions in gradient 3 are shifted with respect to those of gradient 1 and 2. (B) Immunoblot of isopycnic KBr gradients from wild-type or Lpp RNAi hemolymph. Note that LTP shifts to lower-density fractions, when Lpp levels are reduced. (C) Lipid droplets in guts from second instar larvae at different time points after the induction of shibire K44A (dominant negative dynamin) in enterocytes, visualized with Nile red. Within a few hours after induction of shibire K44A, neutral lipid droplets accumulate to a similar extend as in the gut of lipoprotein-deficient larvae. However, guts are almost completely devoid of lipid droplets 24 h after induction. Yellow: neutral lipids; red: phospholipids. Scale bars = 50 µm. See also Figure 4E. (D) Changes in hemolymph DAG upon LTP or Lpp RNAi, quantified by mass spectrometry. DAG species are normalized to hemolymph protein. Lipid species with medium-chain fatty acid residues are indicated by green background. Error bars indicate ±SD (control n = 8; LTP n = 6; Lpp RNAi n = 7; free sterols n = 4. * p<0.05,** p<0.005, *** p<0.0001).
(TIF)

Figure S6 Neutral and polar tissue lipid content. Chain length distribution of fatty acid residues in TAG and DAG species in larval tissues, quantified by mass spectrometry. TAG and DAG species are normalized to polar lipids. Due to the high TAG content of the fat body, TAG levels in gut, wing disc and brain are additionally depicted in a separate panel. Lipid species with medium-chain fatty acid residues are indicated by green background. Error bars indicate ±SD (n = 5).
(TIF)

Figure S7 Lipoproteins are required for the export of sterols from the gut. (A) Unesterified sterols of the posterior midgut, fat body, wing disc and brain of LTP or Lpp RNAi third instar larvae, visualized with Filipin. Knock-down of LTP or Lpp causes a strong reduction in fat body, wing disc and brain sterols, but does not reduce sterols in the gut. Scale bars = 50 µm. (B), (C) Changes in unesterified sterols in (B) gut and (C) fat body upon LTP or Lpp RNAi, quantified by mass spectrometry. Free sterols are normalized to polar lipid. Error bars indicate ±SD (n = 3;* p<0.05).
(TIF)

Figure S8 The role of lipolysis in the mobilization of neutral lipids from the gut. (A) Changes in intestinal TAG species of *bmm* and *akhr* mutant larvae, quantified by mass spectrometry. TAG species are normalized to polar lipid. (B) Lipid droplets in posterior midguts of *bmm* and *akhr* mutant second larvae, visualized with Nile red. Yellow: neutral lipids; red: phospholipids. Scale bars = 20 µm. (C) Changes in hemolymph DAG species of *bmm* and *akhr* mutant larvae, quantified by mass spectrometry. DAG species are normalized to hemolymph protein. (D) Changes in intestinal DAG species of *bmm* and *akhr* mutant larvae, quantified by mass spectrometry. DAG species are normalized to polar lipid. (E) Changes in hemolymph DAG species upon intestinal Bmm RNAi, AKHR RNAi or Bmm+AKHR RNAi, quantified by mass spectrometry. RNAi was driven with MyoIA-GAL4. DAG species are normalized to hemolymph protein. Lipid species with medium-chain fatty acid residues are indicated by green background. Error bars indicate ±SD (n = 3).
(TIF)

Figure S9 The contribution of dietary lipids and intestinal lipogenesis to Lpp medium-chain DAG. (A) Immunoblot of isopycnic KBr gradients of hemolymph prepared from lipid-fed or lipid-starved wild-type larvae, or lipid-fed LTP RNAi larvae. Lipid starvation increases the density of Lpp particles; LTP RNAi increases Lpp density even further. (B) Neutral/polar lipid ratio of hemolymph from lipid-starved and LTP RNAi larvae, quantified by mass spectrometry. Lipid starvation reduces the neutral/polar Lpp lipid ratio. LTP RNAi reduces the neutral/polar Lpp lipid ratio even further. Error bars indicate ±SD (n = 3). (C) Changes in intestinal DAG and TAG species of lipid-fed and lipid-starved Lpp RNAi larvae, quantified by mass spectrometry. Acylglycerols are normalized to polar lipid. Note that Lpp RNAi strongly increases intestinal neutral lipids, regardless the presence of lipids in the diet. Lipid species with medium-chain fatty acid residues are indicated by green background. Error bars indicate ±SD (n = 3). (D) Changes in fat body TAG upon lipid starvation, quantified by mass spectrometry. TAG levels normalized to polar lipid slightly increase in the absence of dietary lipid. Error bars indicate ±SD (n = 5).
(TIF)

Figure S10 Consequences of lipoprotein knock-down on the phospholipid composition of cellular membranes. (A) Changes in hemolymph PE species caused by LTP or Lpp RNAi, quantified by mass spectrometry. Individual PE species are normalized to total hemolymph protein. Note that Lpp RNAi strongly reduces all hemolymph PE species. (B) Changes in PE species in the gut caused by Lpp RNAi, quantified by mass spectrometry. Individual PE species are represented as mol% of polar lipids. PE 32∶1 decreases by about 16%, PE 34∶1 by about 25%. No other PE species decrease significantly. Note in particular that Lpp RNAi does not lower levels of PE 34∶2 and PE 36∶2, which are of similar abundance in the gut as PE 34∶1, but minor constituents of Lpp. (C) Changes in PC species in fat body, gut, wing disc and brain caused by LTP or Lpp RNAi, quantified by mass spectrometry. Individual PC species are represented as mol% of polar lipids. Note that no PC species is significantly decreased in cellular membranes upon either LTP or Lpp RNAi. (D) Plasmalogen PE (PE O-) levels in the brain of LTP and Lpp RNAi animals, quantified by mass spectrometry. Individual plasmalogen PE species are represented as mol% of polar lipids. Note that no plasmalogen PE species is significantly decreased upon either LTP or Lpp RNAi. Error bars indicate ±SD (hemolymph: control n = 8, LTP n = 6, Lpp RNAi n = 7; organs: n = 5).
(TIF)

Figure S11 Supporting mass spectrometry data. (A) Changes in DAG species in hemolymph, fat body, gut, wing disc and brain upon LTP or Lpp RNAi, quantified by mass spectrometry. Shown are both number of double bonds and combined acyl chain length. Hemolymph DAG is normalized to protein; tissue DAG is normalized to polar lipids. Error bars indicate ±SD (hemolymph: control n = 8, LTP n = 6, Lpp RNAi n = 7; organs: n = 5). (B) Changes in TAG species in hemolymph, fat body, gut, wing disc and brain caused by LTP or Lpp RNAi, quantified by mass spectrometry. Shown are both number of double bonds and combined acyl chain length. Hemolymph TAG is normalized to protein; tissue TAG is normalized to polar lipids. Error bars indicate ±SD (hemolymph: control n = 8, LTP n = 6, Lpp RNAi n = 7; organs: n = 5). (C) Total tissue polar lipid count in different LTP and Lpp RNAi mass spectrometry experiments. Similar amounts of a given tissue were extracted and quantified for each condition. Error bars indicate ±SD (n = 5).
(TIF)

Text S1 Supporting Materials and Methods.
(DOCX)

## References

[1] Vance DE, Vance JE (2008). Biochemistry of lipids, lipoproteins and membranes: Elsevier..

[2] Olofsson SO, Asp L, Boren J (1999). The assembly and secretion of apolipoprotein B-containing lipoproteins.. Curr Opin Lipidol.

[3] Hussain MM, Shi J, Dreizen P (2003). Microsomal triglyceride transfer protein and its role in apoB-lipoprotein assembly.. J Lipid Res.

[4] Gregg RE, Wetterau JR (1994). The molecular basis of abetalipoproteinemia.. Curr Opin Lipidol.

[5] Berriot-Varoqueaux N, Aggerbeck LP, Samson-Bouma M, Wetterau JR (2000). The role of the microsomal triglygeride transfer protein in abetalipoproteinemia.. Annu Rev Nutr.

[6] Glass CK, Witztum JL (2001). Atherosclerosis. the road ahead.. Cell.

[7] Eckel RH, Grundy SM, Zimmet PZ (2005). The metabolic syndrome.. Lancet.

[8] Goldstein JL, Brown MS (1977). The low-density lipoprotein pathway and its relation to atherosclerosis.. Annu Rev Biochem.

[9] Spector AA, Mathur SN, Kaduce TL, Hyman BT (1980). Lipid nutrition and metabolism of cultured mammalian cells.. Prog Lipid Res.

[10] Shevchenko A, Simons K (2010). Lipidomics: coming to grips with lipid diversity.. Nat Rev Mol Cell Biol.

[11] Baker KD, Thummel CS (2007). Diabetic larvae and obese flies-emerging studies of metabolism in Drosophila.. Cell Metab.

[12] Leopold P, Perrimon N (2007). Drosophila and the genetics of the internal milieu.. Nature.

[13] Gronke S, Mildner A, Fellert S, Tennagels N, Petry S (2005). Brummer lipase is an evolutionary conserved fat storage regulator in Drosophila.. Cell Metab.

[14] Beller M, Bulankina AV, Hsiao HH, Urlaub H, Jackle H (2010). PERILIPIN-dependent control of lipid droplet structure and fat storage in Drosophila.. Cell Metab.

[15] Birse RT, Choi J, Reardon K, Rodriguez J, Graham S (2010). High-fat-diet-induced obesity and heart dysfunction are regulated by the TOR pathway in Drosophila.. Cell Metab.

[16] Smolenaars MM, Madsen O, Rodenburg KW, Van der Horst DJ (2007). Molecular diversity and evolution of the large lipid transfer protein superfamily.. J Lipid Res.

[17] Herz J, Bock HH (2002). Lipoprotein receptors in the nervous system.. Annu Rev Biochem.

[18] Khaliullina H, Panakova D, Eugster C, Riedel F, Carvalho M (2009). Patched regulates Smoothened trafficking using lipoprotein-derived lipids.. Development.

[19] Parra-Peralbo E, Culi J (2011). Drosophila lipophorin receptors mediate the uptake of neutral lipids in oocytes and imaginal disc cells by an endocytosis-independent mechanism.. PLoS Genet.

[20] Eugster C, Panakova D, Mahmoud A, Eaton S (2007). Lipoprotein-heparan sulfate interactions in the Hh pathway.. Dev Cell.

[21] MacArthur JM, Bishop JR, Stanford KI, Wang L, Bensadoun A (2007). Liver heparan sulfate proteoglycans mediate clearance of triglyceride-rich lipoproteins independently of LDL receptor family members.. J Clin Invest.

[22] Ryan RO, Van der Horst DJ (2000). Lipid transport biochemistry and its role in energy production.. Annu Rev Entomol.

[23] Canavoso LE, Jouni ZE, Karnas KJ, Pennington JE, Wells MA (2001). Fat metabolism in insects.. Annu Rev Nutr.

[24] Prasad SV, Fernando-Warnakulasuriya GJ, Sumida M, Law JH, Wells MA (1986). Lipoprotein biosynthesis in the larvae of the tobacco hornworm, Manduca sexta.. J Biol Chem.

[25] Arrese EL, Soulages JL (2010). Insect fat body: energy, metabolism, and regulation.. Annu Rev Entomol.

[26] Canavoso LE, Wells MA (2001). Role of lipid transfer particle in delivery of diacylglycerol from midgut to lipophorin in larval Manduca sexta.. Insect Biochem Mol Biol.

[27] Canavoso LE, Yun HK, Jouni ZE, Wells MA (2004). Lipid transfer particle mediates the delivery of diacylglycerol from lipophorin to fat body in larval Manduca sexta.. J Lipid Res.

[28] Van Heusden MC, Law JH (1989). An insect lipid transfer particle promotes lipid loading from fat body to lipoprotein.. J Biol Chem.

[29] Panakova D, Sprong H, Marois E, Thiele C, Eaton S (2005). Lipoprotein particles are required for Hedgehog and Wingless signalling.. Nature.

[30] Smolenaars MM, de Morree A, Kerver J, Van der Horst DJ, Rodenburg KW (2007). Insect lipoprotein biogenesis depends on an amphipathic beta cluster in apolipophorin II/I and is stimulated by microsomal triglyceride transfer protein.. J Lipid Res.

[31] Sellers JA, Hou L, Athar H, Hussain MM, Shelness GS (2003). A Drosophila microsomal triglyceride transfer protein homolog promotes the assembly and secretion of human apolipoprotein B. Implications for human and insect transport and metabolism.. J Biol Chem.

[32] Shoulders CC, Narcisi TM, Read J, Chester A, Brett DJ (1994). The abetalipoproteinemia gene is a member of the vitellogenin family and encodes an alpha-helical domain.. Nat Struct Biol.

[33] Babin PJ, Bogerd J, Kooiman FP, Van Marrewijk WJ, Van der Horst DJ (1999). Apolipophorin II/I, apolipoprotein B, vitellogenin, and microsomal triglyceride transfer protein genes are derived from a common ancestor.. J Mol Evol.

[34] Kutty RK, Kutty G, Kambadur R, Duncan T, Koonin EV (1996). Molecular characterization and developmental expression of a retinoid- and fatty acid-binding glycoprotein from Drosophila. A putative lipophorin.. J Biol Chem.

[35] Ryan RO, Wells MA, Law JH (1986). Lipid transfer protein from Manduca sexta hemolymph.. Biochem Biophys Res Commun.

[36] Hirayama Y, Chino H (1990). Lipid transfer particle in locust hemolymph: purification and characterization.. J Lipid Res.

[37] Chen J, Honeyager SM, Schleede J, Avanesov A, Laughon A (2012). Crossveinless d is a vitellogenin-like lipoprotein that binds BMPs and HSPGs, and is required for normal BMP signaling in the Drosophila wing.. Development.

[38] Ryan RO, van der Horst DJ (2000). Lipid transport biochemistry and its role in energy production.. Annu Rev Entomol.

[39] Banaszak L, Sharrock W, Timmins P (1991). Structure and function of a lipoprotein: lipovitellin.. Annu Rev Biophys Biophys Chem.

[40] Shibata Y, Branicky R, Landaverde IO, Hekimi S (2003). Redox regulation of germline and vulval development in Caenorhabditis elegans.. Science.

[41] Sellers JA, Hou L, Schoenberg DR, Batistuzzo de Medeiros SR, Wahli W (2005). Microsomal triglyceride transfer protein promotes the secretion of Xenopus laevis vitellogenin A1.. J Biol Chem.

[42] Bownes M (1992). Why is there sequence similarity between insect yolk proteins and vertebrate lipases?. J Lipid Res.

[43] Blacklock BJ, Smillie M, Ryan RO (1992). Insect lipid transfer particle can facilitate net vectorial lipid transfer via a carrier-mediated mechanism.. J Biol Chem.

[44] Gronke S, Muller G, Hirsch J, Fellert S, Andreou A (2007). Dual lipolytic control of body fat storage and mobilization in Drosophila.. PLoS Biol.

[45] de Renobales M, Blomquist GJ (1984). Biosynthesis of medium chain fatty acids in Drosophila melanogaster.. Arch Biochem Biophys.

[46] Brankatschk M, Eaton S (2010). Lipoprotein particles cross the blood-brain barrier in Drosophila.. J Neurosci.

[47] Gordon DA, Jamil H, Gregg RE, Olofsson SO, Boren J (1996). Inhibition of the microsomal triglyceride transfer protein blocks the first step of apolipoprotein B lipoprotein assembly but not the addition of bulk core lipids in the second step.. J Biol Chem.

[48] Raabe M, Veniant MM, Sullivan MA, Zlot CH, Bjorkegren J (1999). Analysis of the role of microsomal triglyceride transfer protein in the liver of tissue-specific knockout mice.. J Clin Invest.

[49] Wang Y, Tran K, Yao Z (1999). The activity of microsomal triglyceride transfer protein is essential for accumulation of triglyceride within microsomes in McA-RH7777 cells. A unified model for the assembly of very low density lipoproteins.. J Biol Chem.

[50] Kulinski A, Rustaeus S, Vance JE (2002). Microsomal triacylglycerol transfer protein is required for lumenal accretion of triacylglycerol not associated with ApoB, as well as for ApoB lipidation.. J Biol Chem.

[51] Rava P, Ojakian GK, Shelness GS, Hussain MM (2006). Phospholipid transfer activity of microsomal triacylglycerol transfer protein is sufficient for the assembly and secretion of apolipoprotein B lipoproteins.. J Biol Chem.

[52] Ryan RO, Senthilathipan KR, Wells MA, Law JH (1988). Facilitated diacylglycerol exchange between insect hemolymph lipophorins. Properties of Manduca sexta lipid transfer particle.. J Biol Chem.

[53] Ryan RO, Wessler AN, Price HM, Ando S, Yokoyama S (1990). Insect lipid transfer particle catalyzes bidirectional vectorial transfer of diacylglycerol from lipophorin to human low density lipoprotein.. J Biol Chem.

[54] Silver ET, Scraba DG, Ryan RO (1990). Lipid transfer particle-induced transformation of human high density lipoprotein into apolipoprotein A-I-deficient low density particles.. J Biol Chem.

[55] Mullaney BC, Ashrafi K (2009). C. elegans fat storage and metabolic regulation.. Biochim Biophys Acta.

[56] Hsieh J, Hayashi AA, Webb J, Adeli K (2008). Postprandial dyslipidemia in insulin resistance: mechanisms and role of intestinal insulin sensitivity.. Atheroscler.

[57] Minahk C, Kim KW, Nelson R, Trigatti B, Lehner R (2008). Conversion of low density lipoprotein-associated phosphatidylcholine to triacylglycerol by primary hepatocytes.. J Biol Chem.

[58] Robichaud JC, van der Veen JN, Yao Z, Trigatti B, Vance DE (2009). Hepatic uptake and metabolism of phosphatidylcholine associated with high density lipoproteins.. Biochim Biophys Acta.

[59] Carvalho M, Schwudke D, Sampaio JL, Palm W, Riezman I (2010). Survival strategies of a sterol auxotroph.. Development.

[60] Horton JD, Goldstein JL, Brown MS (2002). SREBPs: activators of the complete program of cholesterol and fatty acid synthesis in the liver.. J Clin Invest.

[61] Nohturfft A, Zhang SC (2009). Coordination of lipid metabolism in membrane biogenesis.. Annu Rev Cell Dev Biol.

[62] Dobrosotskaya IY, Seegmiller AC, Brown MS, Goldstein JL, Rawson RB (2002). Regulation of SREBP processing and membrane lipid production by phospholipids in Drosophila.. Science.

[63] Shapiro JP, Law JH (1983). Locust adipokinetic hormone stimulates lipid mobilization in Manduca sexta.. Biochem Biophys Res Commun.

[64] Sandhoff R, Brugger B, Jeckel D, Lehmann WD, Wieland FT (1999). Determination of cholesterol at the low picomole level by nano-electrospray ionization tandem mass spectrometry.. J Lipid Res.

