# Supplementary figures and images for: Lipoproteins in Drosophila melanogaster—Assembly, Function, and Influence on Tissue Lipid Composition
Source: PLoS Genet. 2012 Jul 26;8(7):e1002828. doi: 10.1371/journal.pgen.1002828 (PMC3406001; doi:10.1371/journal.pgen.1002828)

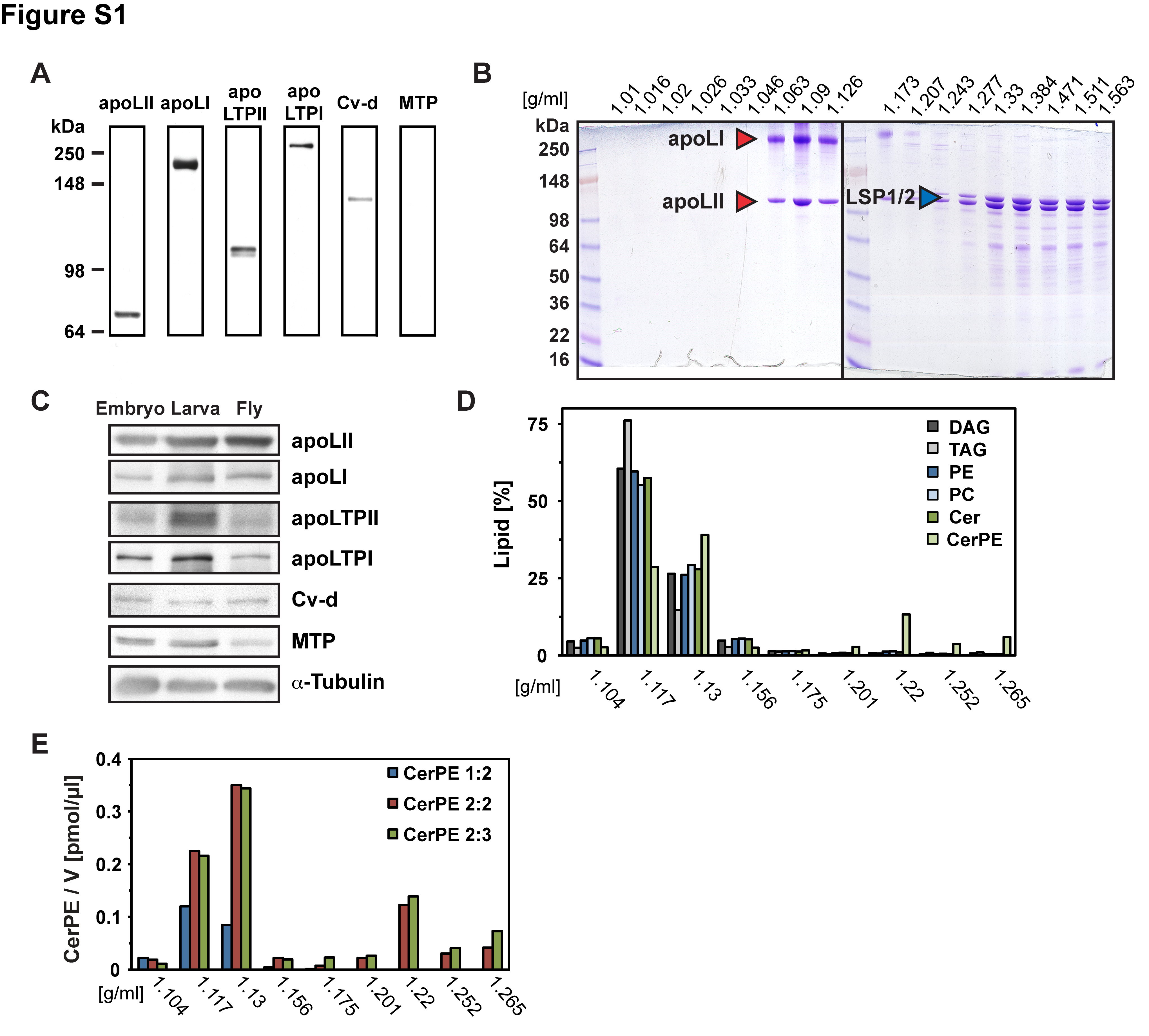

Supplement: Figure S1 — Drosophila lipoproteins and their lipid content. (A) Specificity of apolipoprotein antibodies. An immunoblot of hemolymph from feeding third instar larvae was consecutively probed for the individual proteins. The apoLTPII antibody recognizes two proteins of similar molecular weight. ApoLTP harbors two closely spaced furin consensus sequences (see Figure 1A), indicating that the two forms of apoLTPII are generated by alternative use of either cleavage site. Note that MTP likewise exists in two isoforms of similar size (see Figure 2D, 2E); however, we could not identify putative protease consensus sites in the MTP sequence. (B) Coomassie staining of hemolymph proteins fractionated on an isopycnic Optiprep gradient. Lpp is the only detectable protein present in the lower-density fractions 1–9. Note that NuPAGE MES buffer was used for electrophoresis of this gradient, whereas electrophoresis of the gradients shown in Figure 1C, 1D was performed with Tris-glycine buffer. (C) Immunoblot showing that apoLpp, apoLTP, Cv-d and MTP proteins are present in embryos, third instar larvae and adult flies. (D) Distribution of lipid classes in the hemolymph density gradient from Figure 1E. Shown is the % lipid of each lipid class present in each fraction. (E) Ceramide-Phosphorylethanolamine (CerPE) present in different fractions of the hemolymph density gradient of Figure 1E. Shown is the total amount of CerPE present in each gradient fraction from 1 µl hemolymph. Note that CerPE 2∶2 and 2∶3 (2 double bonds, 2 or 3 hydroxyl groups) partially co-fractionate with LTP, whereas CerPE 1∶2 (1 double bond, 2 hydroxyl groups) is confined to the Lpp fractions. (TIF) [file pgen.1002828.s001.tif]

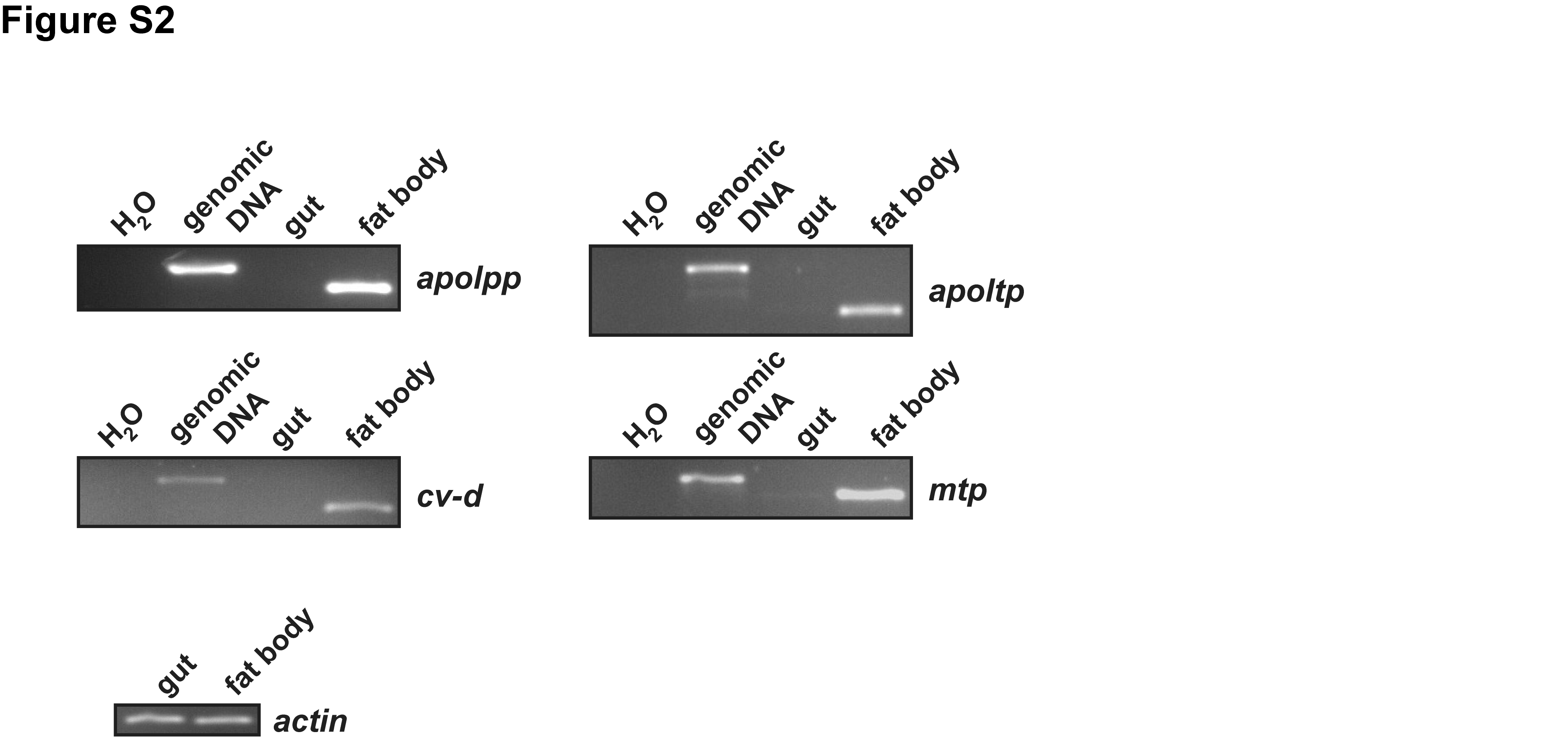

Supplement: Figure S2 — Apolipoprotein transcripts are not detectable in the gut. Reverse transcription PCR showing that apolipoprotein transcripts can be detected in the fat body, but not in the gut of third instar larvae. Primer pairs were designed to span small introns to preclude contamination with genomic DNA. Note that actin transcripts can be readily detected in cDNA preparations of both fat body and gut. (TIF) [file pgen.1002828.s002.tif]

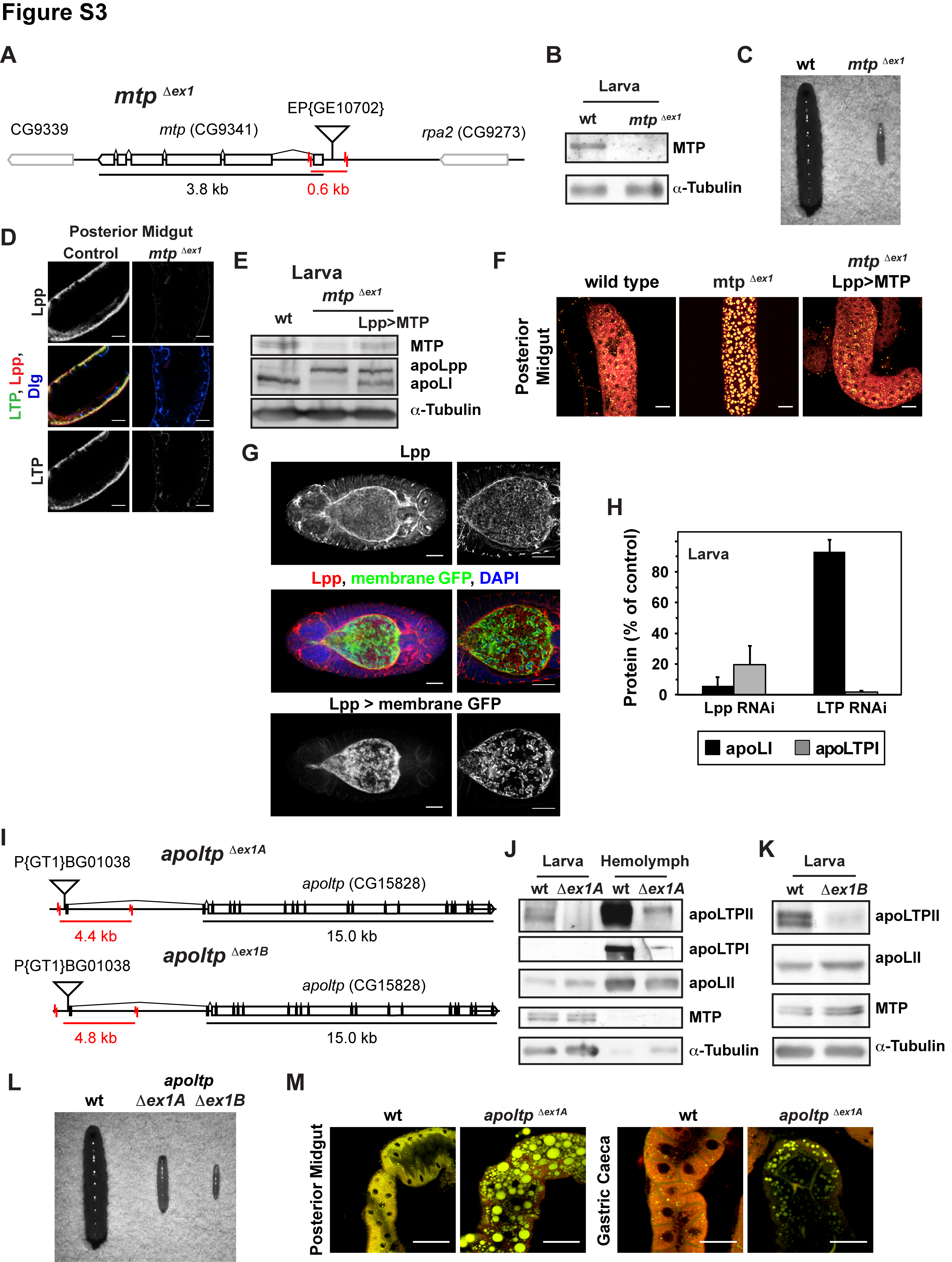

Supplement: Figure S3 — Phenotypes of apolpp, apoltp and mtp mutants. (A) Schematic representation of the mtp null allele mtpΔex1. (B) Immunoblot of larvae homozygous for mtpΔex1. Mutant animals lack any detectable MTP protein. (C) Wild-type and mtpΔex1 larvae 4 days after egg laying. Wild-type animals have reached the third larval instar. Animals homozygous for mtpΔex1 arrest in the first larval instar. (D) Immunofluorescence of the posterior midgut from first instar mtpΔex1 mutant larvae showing that mtp mutant guts lack detectable Lpp and LTP. Basolateral membranes are marked with discs large (Dlg). Scale bars = 20 µm. (E) Immunblot of first instar mtpΔex1 larvae in which lipoprotein production was rescued by fat body-specific expression of MTP with Lpp-GAL4. ApoLpp cleavage is impaired in mtpΔex1 larvae, but restored through fat body-specific expression of MTP. (F) Fat body-specific expression of MTP with Lpp-GAL4 in mtpΔex1 larvae rescues intestinal lipid mobilization. Lipid droplets of first instar posterior midguts are visualized with Nile red. Yellow: neutral lipids, red: phospholipids. Scale bars = 20 µm. (G) Immunofluorescence showing that Lpp is produced in yolk cells of stage 14 embryos, but then spreads throughout the whole embryo. Lpp expression is visualized with Lpp-GAL4-driven membrane GFP (CD8-GFP). Nuclei are visualized with DAPI. Scale bars = 50 µm. (H) Knock-down efficiency of Lpp and LTP in third instar larvae, 4 days after induction of RNAi. ApoLI and apoLTPI levels in whole larval extracts were quantified by immunoblotting. Note that Lpp RNAi entails a concomitant partial reduction of LTP. Error bars indicate ±SD (n = 5). (I) Schematic representation of the apoltp alleles apoltpΔex1A and apoltpΔex1B. (J) Immunoblot of second instar larvae homozygous for apoltpΔex1A and their hemolymph. Mutant animals show strongly reduced apoLTPI and apoLTPII levels, with apoLII and MTP being unaffected. (K) Immunoblot of first instar larvae homozygous for apoltpΔex1B. Mutant animal [file pgen.1002828.s003.tif]

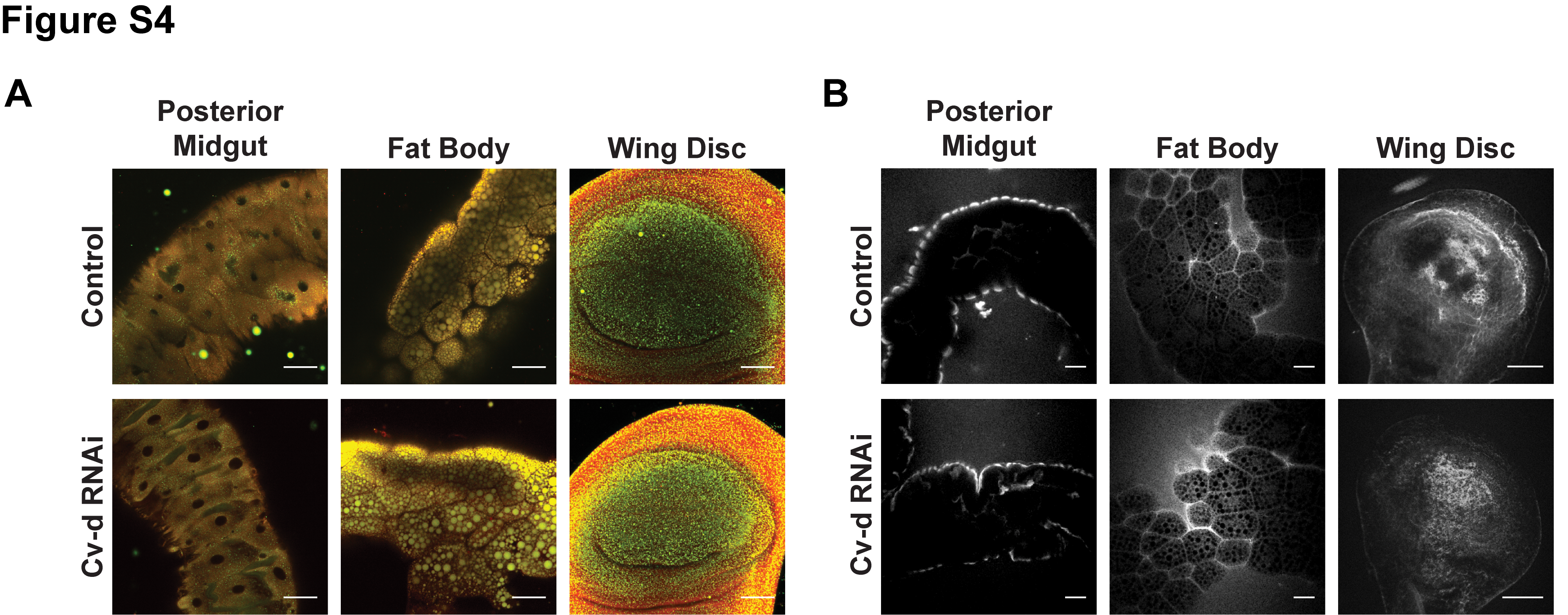

Supplement: Figure S4 — Phenotypes of RNAi against the vitellogenin-like protein Cv-d. (A) Lipid droplets in the posterior midgut, fat body and wing disc of Cv-d RNAi third instar larvae visualized with Nile red. Yellow: neutral lipids; red: phospholipids. Cv-d knock-down does not obviously perturb lipid droplets in any organ. Scale bars = 50 µm. (B) Unesterified sterols of the gut, fat body and wing disc of Cv-d RNAi third instar larvae visualized with Filipin. Sterols can still be detected in all organs upon Cv-d knock-down. Scale bars = 50 µm. (TIF) [file pgen.1002828.s004.tif]

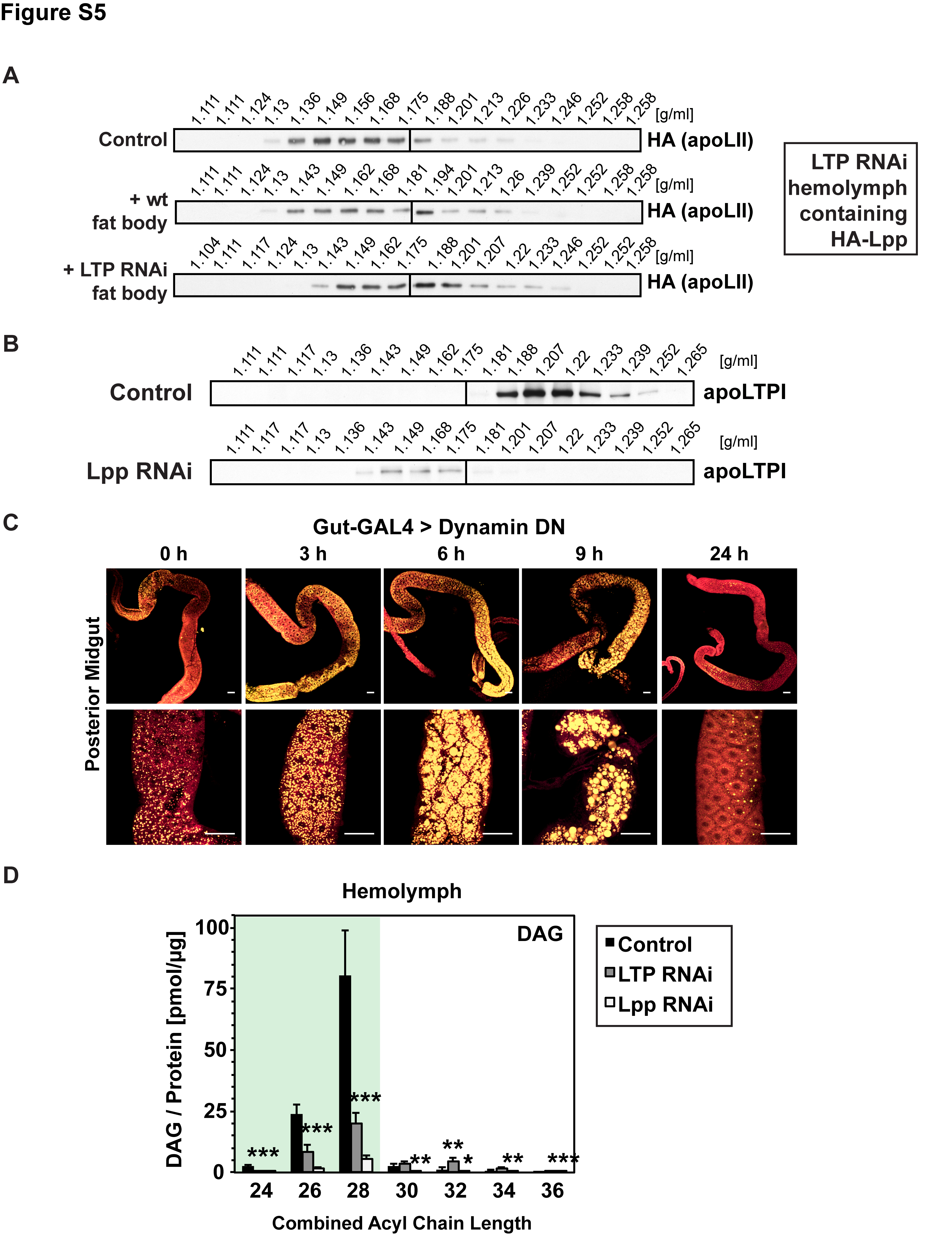

Supplement: Figure S5 — Lipid mobilization from fat body and gut. (A) Immunoblot of isopycnic KBr gradients from lipid transfer experiments between fat bodies and LTP RNAi hemolymph containing HA-Lpp. Lpp density does not decrease upon incubation with fat bodies, regardless the presence of LTP. Note that the density of fractions in gradient 3 are shifted with respect to those of gradient 1 and 2. (B) Immunoblot of isopycnic KBr gradients from wild-type or Lpp RNAi hemolymph. Note that LTP shifts to lower-density fractions, when Lpp levels are reduced. (C) Lipid droplets in guts from second instar larvae at different time points after the induction of shibire K44A (dominant negative dynamin) in enterocytes, visualized with Nile red. Within a few hours after induction of shibire K44A, neutral lipid droplets accumulate to a similar extend as in the gut of lipoprotein-deficient larvae. However, guts are almost completely devoid of lipid droplets 24 h after induction. Yellow: neutral lipids; red: phospholipids. Scale bars = 50 µm. See also Figure 4E. (D) Changes in hemolymph DAG upon LTP or Lpp RNAi, quantified by mass spectrometry. DAG species are normalized to hemolymph protein. Lipid species with medium-chain fatty acid residues are indicated by green background. Error bars indicate ±SD (control n = 8; LTP n = 6; Lpp RNAi n = 7; free sterols n = 4. * p<0.05,** p<0.005, *** p<0.0001). (TIF) [file pgen.1002828.s005.tif]

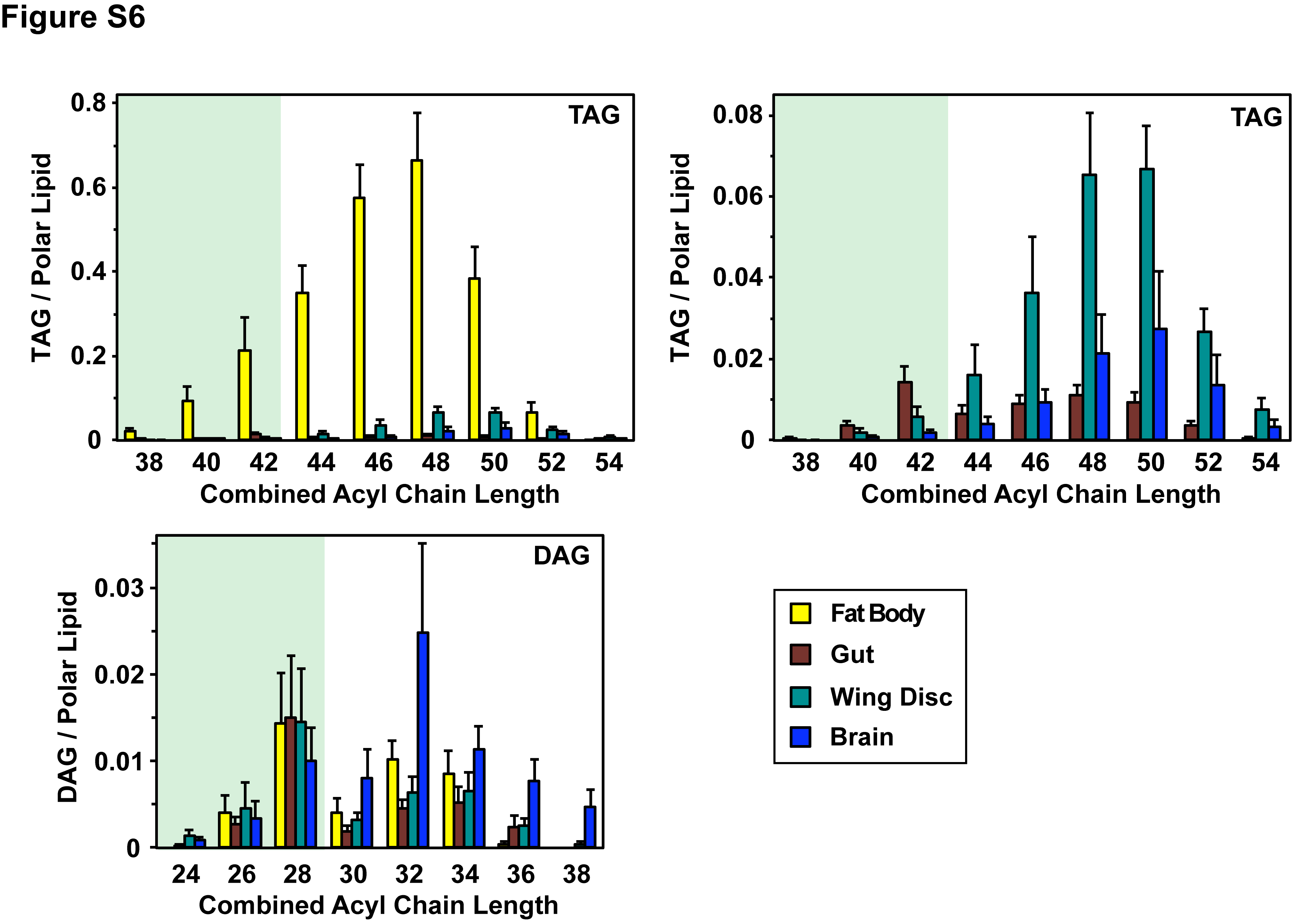

Supplement: Figure S6 — Neutral and polar tissue lipid content. Chain length distribution of fatty acid residues in TAG and DAG species in larval tissues, quantified by mass spectrometry. TAG and DAG species are normalized to polar lipids. Due to the high TAG content of the fat body, TAG levels in gut, wing disc and brain are additionally depicted in a separate panel. Lipid species with medium-chain fatty acid residues are indicated by green background. Error bars indicate ±SD (n = 5). (TIF) [file pgen.1002828.s006.tif]

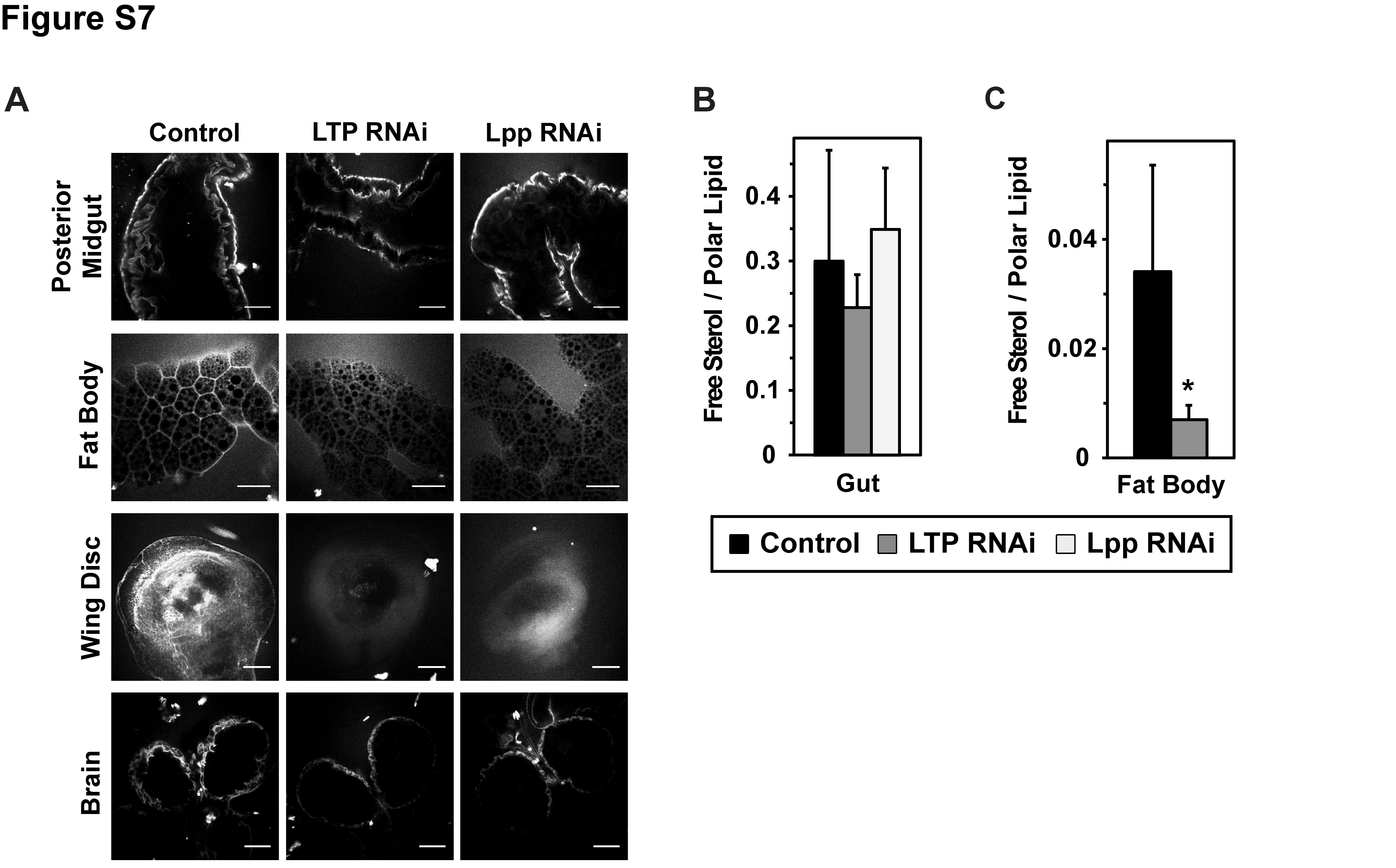

Supplement: Figure S7 — Lipoproteins are required for the export of sterols from the gut. (A) Unesterified sterols of the posterior midgut, fat body, wing disc and brain of LTP or Lpp RNAi third instar larvae, visualized with Filipin. Knock-down of LTP or Lpp causes a strong reduction in fat body, wing disc and brain sterols, but does not reduce sterols in the gut. Scale bars = 50 µm. (B), (C) Changes in unesterified sterols in (B) gut and (C) fat body upon LTP or Lpp RNAi, quantified by mass spectrometry. Free sterols are normalized to polar lipid. Error bars indicate ±SD (n = 3;* p<0.05). (TIF) [file pgen.1002828.s007.tif]

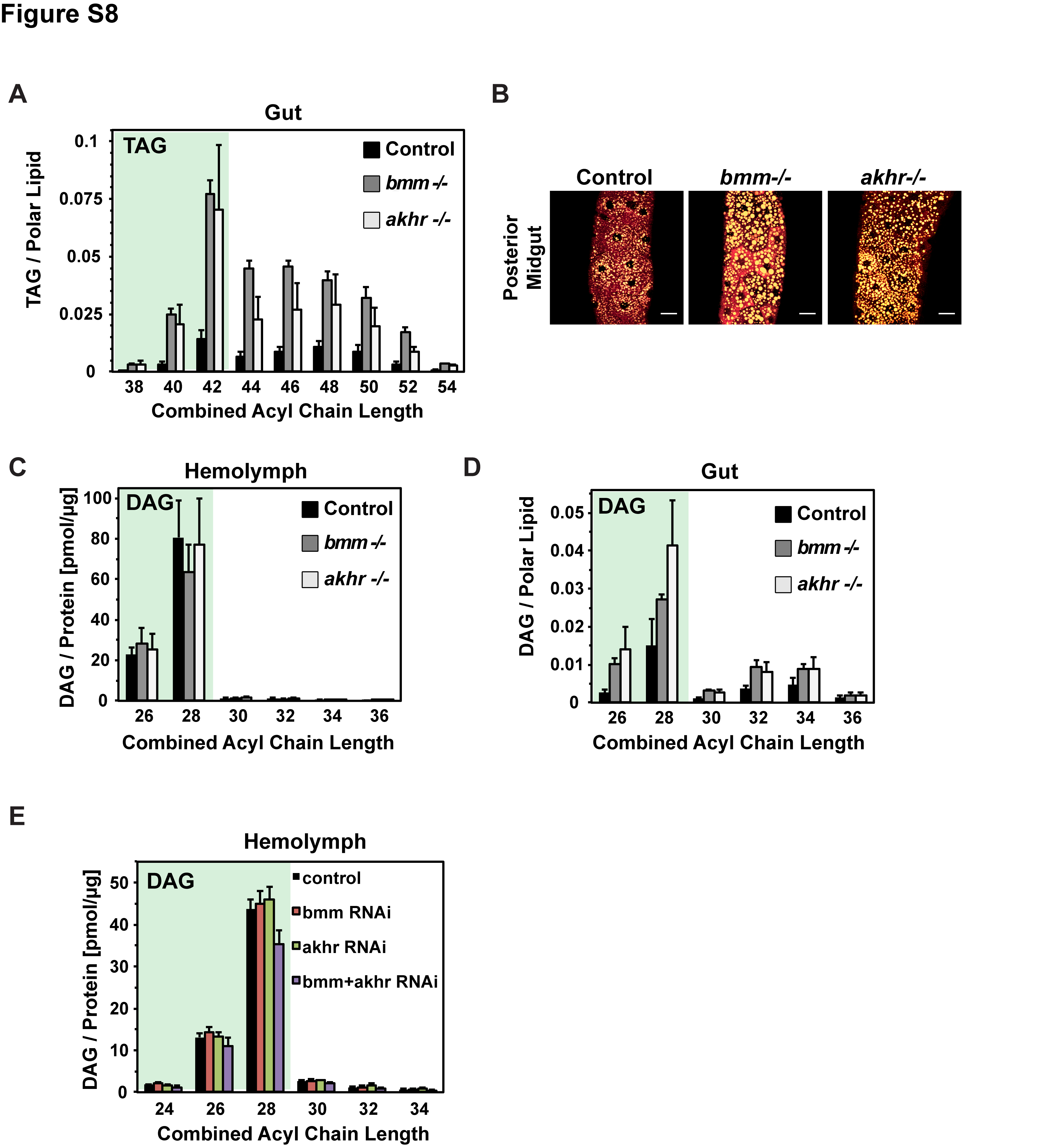

Supplement: Figure S8 — The role of lipolysis in the mobilization of neutral lipids from the gut. (A) Changes in intestinal TAG species of bmm and akhr mutant larvae, quantified by mass spectrometry. TAG species are normalized to polar lipid. (B) Lipid droplets in posterior midguts of bmm and akhr mutant second larvae, visualized with Nile red. Yellow: neutral lipids; red: phospholipids. Scale bars = 20 µm. (C) Changes in hemolymph DAG species of bmm and akhr mutant larvae, quantified by mass spectrometry. DAG species are normalized to hemolymph protein. (D) Changes in intestinal DAG species of bmm and akhr mutant larvae, quantified by mass spectrometry. DAG species are normalized to polar lipid. (E) Changes in hemolymph DAG species upon intestinal Bmm RNAi, AKHR RNAi or Bmm+AKHR RNAi, quantified by mass spectrometry. RNAi was driven with MyoIA-GAL4. DAG species are normalized to hemolymph protein. Lipid species with medium-chain fatty acid residues are indicated by green background. Error bars indicate ±SD (n = 3). (TIF) [file pgen.1002828.s008.tif]

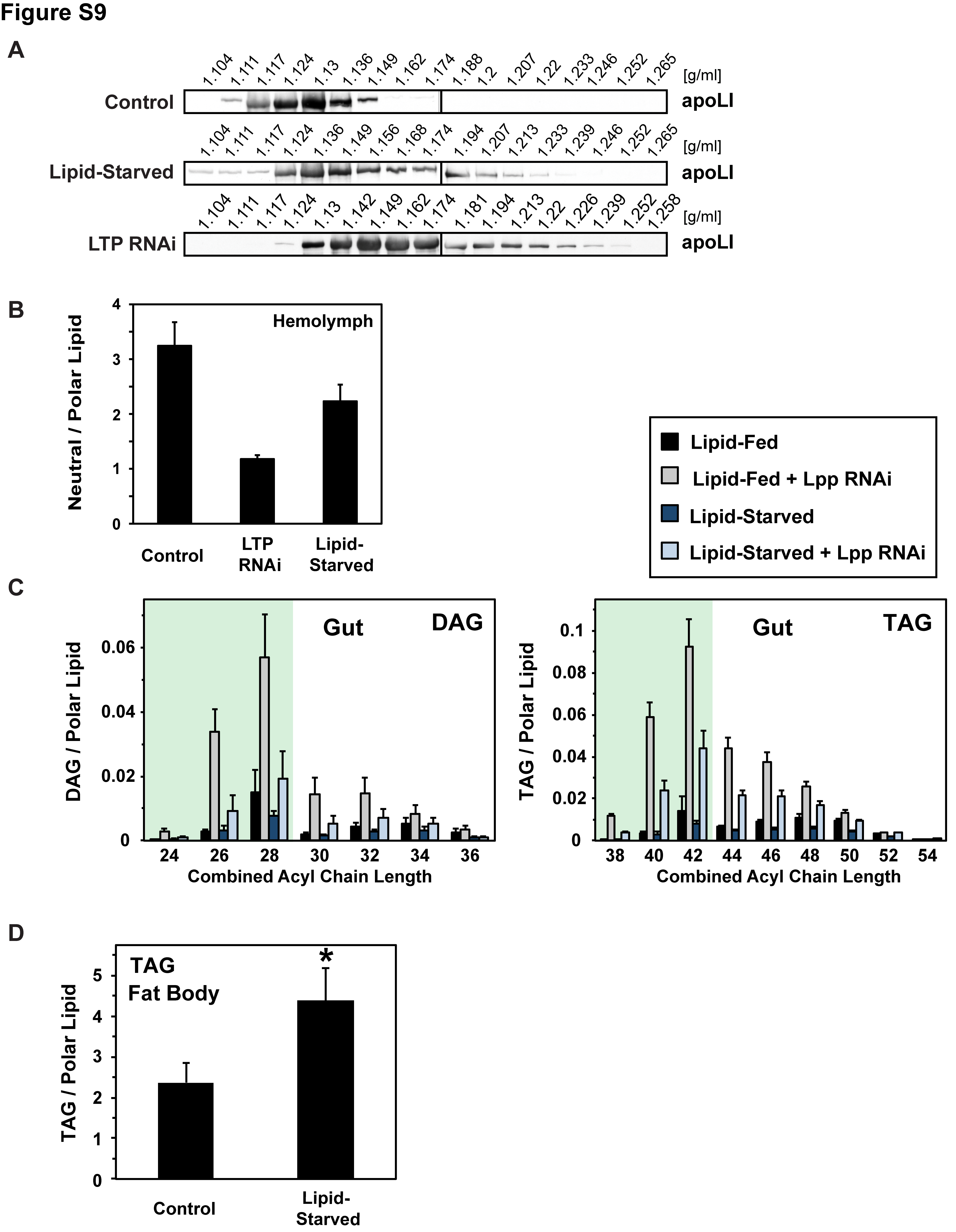

Supplement: Figure S9 — The contribution of dietary lipids and intestinal lipogenesis to Lpp medium-chain DAG. (A) Immunoblot of isopycnic KBr gradients of hemolymph prepared from lipid-fed or lipid-starved wild-type larvae, or lipid-fed LTP RNAi larvae. Lipid starvation increases the density of Lpp particles; LTP RNAi increases Lpp density even further. (B) Neutral/polar lipid ratio of hemolymph from lipid-starved and LTP RNAi larvae, quantified by mass spectrometry. Lipid starvation reduces the neutral/polar Lpp lipid ratio. LTP RNAi reduces the neutral/polar Lpp lipid ratio even further. Error bars indicate ±SD (n = 3). (C) Changes in intestinal DAG and TAG species of lipid-fed and lipid-starved Lpp RNAi larvae, quantified by mass spectrometry. Acylglycerols are normalized to polar lipid. Note that Lpp RNAi strongly increases intestinal neutral lipids, regardless the presence of lipids in the diet. Lipid species with medium-chain fatty acid residues are indicated by green background. Error bars indicate ±SD (n = 3). (D) Changes in fat body TAG upon lipid starvation, quantified by mass spectrometry. TAG levels normalized to polar lipid slightly increase in the absence of dietary lipid. Error bars indicate ±SD (n = 5). (TIF) [file pgen.1002828.s009.tif]

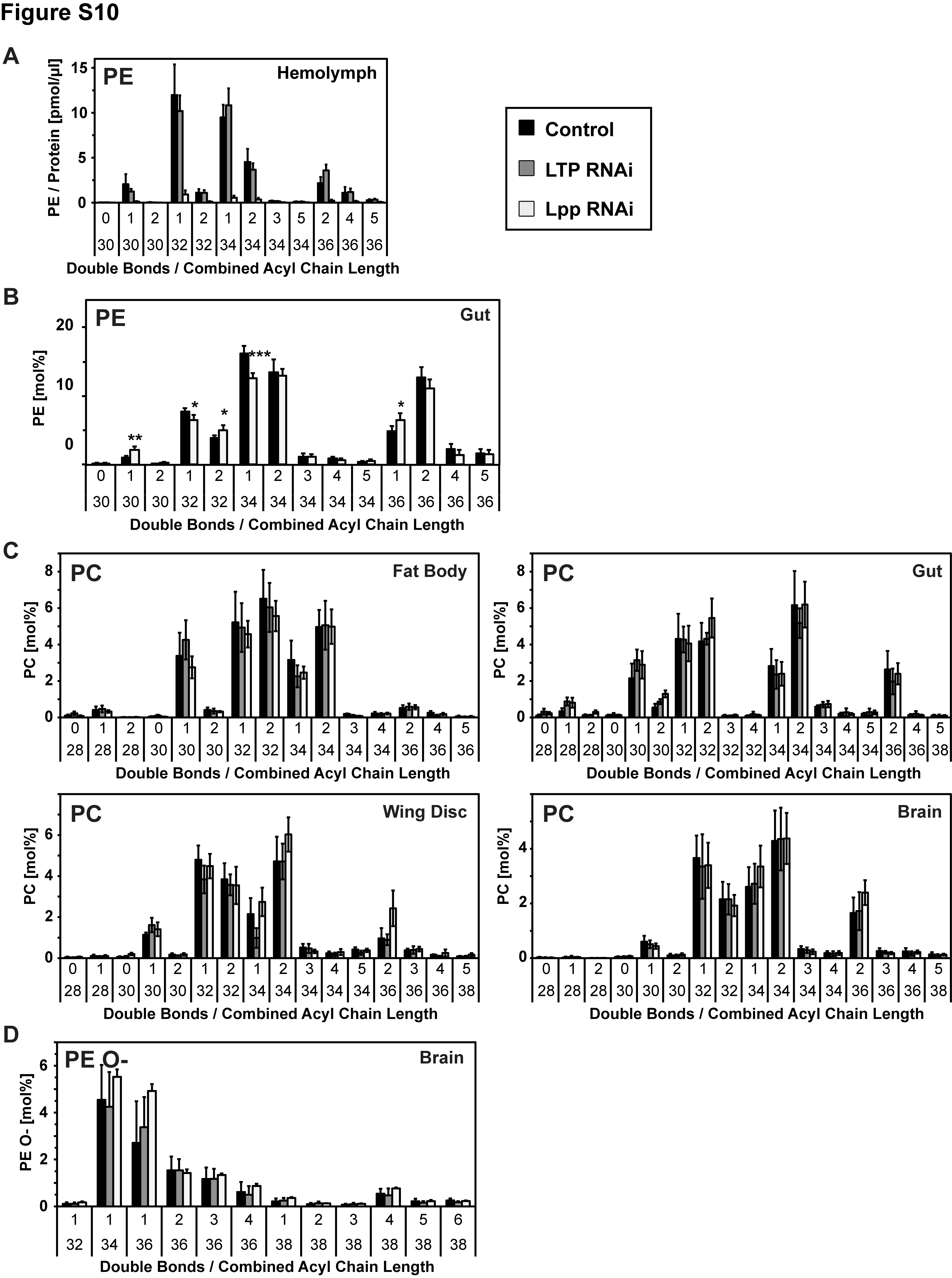

Supplement: Figure S10 — Consequences of lipoprotein knock-down on the phospholipid composition of cellular membranes. (A) Changes in hemolymph PE species caused by LTP or Lpp RNAi, quantified by mass spectrometry. Individual PE species are normalized to total hemolymph protein. Note that Lpp RNAi strongly reduces all hemolymph PE species. (B) Changes in PE species in the gut caused by Lpp RNAi, quantified by mass spectrometry. Individual PE species are represented as mol% of polar lipids. PE 32∶1 decreases by about 16%, PE 34∶1 by about 25%. No other PE species decrease significantly. Note in particular that Lpp RNAi does not lower levels of PE 34∶2 and PE 36∶2, which are of similar abundance in the gut as PE 34∶1, but minor constituents of Lpp. (C) Changes in PC species in fat body, gut, wing disc and brain caused by LTP or Lpp RNAi, quantified by mass spectrometry. Individual PC species are represented as mol% of polar lipids. Note that no PC species is significantly decreased in cellular membranes upon either LTP or Lpp RNAi. (D) Plasmalogen PE (PE O-) levels in the brain of LTP and Lpp RNAi animals, quantified by mass spectrometry. Individual plasmalogen PE species are represented as mol% of polar lipids. Note that no plasmalogen PE species is significantly decreased upon either LTP or Lpp RNAi. Error bars indicate ±SD (hemolymph: control n = 8, LTP n = 6, Lpp RNAi n = 7; organs: n = 5). (TIF) [file pgen.1002828.s010.tif]

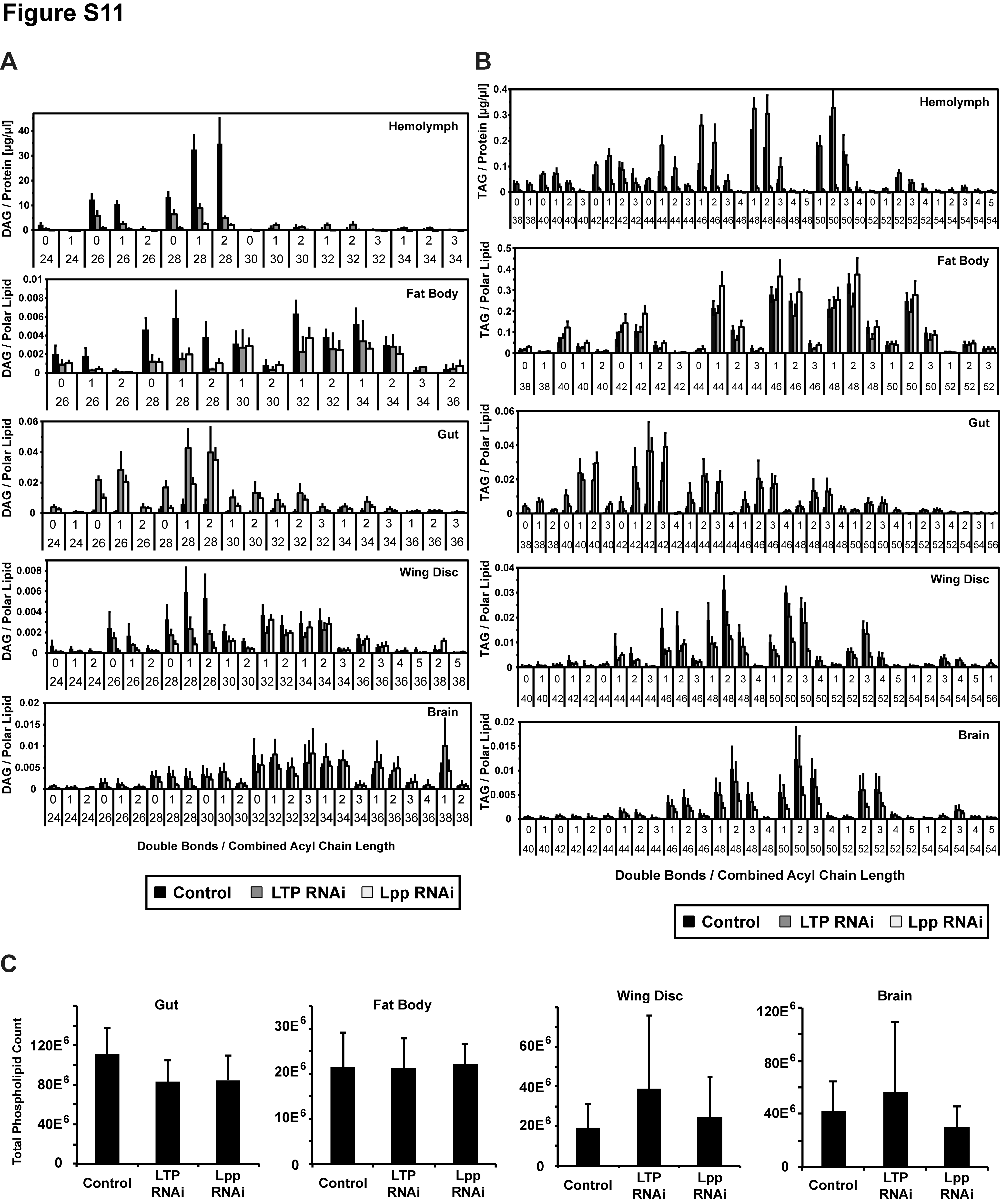

Supplement: Figure S11 — Supporting mass spectrometry data. (A) Changes in DAG species in hemolymph, fat body, gut, wing disc and brain upon LTP or Lpp RNAi, quantified by mass spectrometry. Shown are both number of double bonds and combined acyl chain length. Hemolymph DAG is normalized to protein; tissue DAG is normalized to polar lipids. Error bars indicate ±SD (hemolymph: control n = 8, LTP n = 6, Lpp RNAi n = 7; organs: n = 5). (B) Changes in TAG species in hemolymph, fat body, gut, wing disc and brain caused by LTP or Lpp RNAi, quantified by mass spectrometry. Shown are both number of double bonds and combined acyl chain length. Hemolymph TAG is normalized to protein; tissue TAG is normalized to polar lipids. Error bars indicate ±SD (hemolymph: control n = 8, LTP n = 6, Lpp RNAi n = 7; organs: n = 5). (C) Total tissue polar lipid count in different LTP and Lpp RNAi mass spectrometry experiments. Similar amounts of a given tissue were extracted and quantified for each condition. Error bars indicate ±SD (n = 5). (TIF) [file pgen.1002828.s011.tif]
