# Supplementary material for: Lipoproteins in Drosophila melanogaster—Assembly, Function, and Influence on Tissue Lipid Composition
Source: PLoS Genet. 2012 Jul 26;8(7):e1002828. doi: 10.1371/journal.pgen.1002828 (PMC3406001; doi:10.1371/journal.pgen.1002828)
Supplement: Text S1 — Supporting Materials and Methods. (DOCX) [file pgen.1002828.s012.docx]

**Palm et al. Lipoproteins in *Drosophila melanogaster* – Assembly, Function, and Influence on Tissue Lipid Composition**

**Supporting Materials and Methods**

**Fly Stocks**
Wild type Oregon R, a PiggyBac *apolpp* mutant, UAS-Shibire K44A, Adh-GAL4, Tub-GAL80^TS^, Tub-GAL4 lines, and p-element lines used for the generation of *mtp* and *apoltp* mutants were obtained from Bloomington. CG31150 (Cv-d) RNAi, FAS RNAi, Bmm RNAi, AKHR RNAi and UAS-Dicer2 lines were obtained from VDRC. Lpp-GAL4 and HA-tagged apoLpp were described in Brankatschk and Eaton, 2010. *bmm* and *akhr* mutants were a kind gift of Ronald Kühnlein. MyoIA-GAL4 was a kind gift of Bruce Edgar. We note that the driver MyoIA-GAL4 itself causes strong accumulation of DAG and TAG in the gut.

**Generation of *mtp* and *apoltp* Mutants**

A deletions in *mtp* was generated by imprecise excision of EP[GE10702]. Deletions in *apoltp* were generated by imprecise excision of P[GT1]BG01038. Lines were screened for larval lethality; the lesion of the lethal lines was defined by PCR. One *mtp* and two *apoltp* deletion alleles were obtained this way: *mtp^Δex1^* harbors a 559 bp deletion. *apoltp^Δex1A^* harbors a 4980 bp deletion; *apoltpDex1C* harbors a 4364 bp. All these alleles lack the first exon including the translational start site.

Homozygous *mtp^Δex1^* animals arrest in the first larval instar. Development to adulthood can be rescued by fat body specific expression of UAS-MTP with Lpp-GAL4. Animals homozygous for *apoltp^Δex1A^* arrest in the second larval instar: animals homozygous for *apoltp^Δex1B^* arrest in the first larval instar. Trans-heterozygous *apoltp^Δex1A^* / *apoltp^Δex1B^* animals similarly arrest development, as do animals trans-heterozygous for *apoltp^Δex1A^* or *apoltp^Δex1B^* and the deficiency Df(2L)Exel6024 (available from the Bloomington Stock Center), which spans 30C1 – 30C9 including the *apoltp* locus. An *apoltp* rescue construct was generated by phiC31-mediated integration of a fosmid containing the genomic region 2L:9632297 – 9670685 [[1](#_ENREF_1)], which includes the *apoltp* locus, into the attP2 landing site [[2](#_ENREF_2)]. This fosmid rescues the development of animals homozygous for *apoltp^Δex1A^* or *apoltp^Δex1B^* to fertile adult flies.

**Generation of RNAi Constructs**
Transgenic flies harboring inducible RNAi constructs (UAS<HcRed>dsRNA) targeting the mRNA of *apoltp* or *mtp* were generated using pFRiPE, in which RNAi is initiated by excision of a FLP cassette [[3](#_ENREF_3)]. Repeat units corresponding to nucleotides 425 – 944 and 11525 – 12082 of CG15828 were used for targeting sequences corresponding to apoLTPII and apoLTPI, respectively. A repeat unit corresponding to nucleotides 67 - 746 of CG9342 was used for targeting MTP.
Inducible *apolpp* RNAi transgenics targeting apoLII and apoLI were described in [[4](#_ENREF_4)]. RNAi efficiency was increased by introduction of UAS-Dicer2.
Lpp RNAi also entails a reduction of LTP in all organs including the hemolymph. This is unlikely due to an off-target effect, as both Lpp RNAi constructs similarly decrease LTP, suggesting decreased stability or transcriptional down-regulation. Note that the huge differences between Lpp and LTP in terms of both abundance and lipid content allow for unambiguous interpretation of most of the data presented here. Neither Lpp RNAi nor LTP RNAi affects MTP or Cv-d levels.

**Antibodies**
ApoLTPII and apoLTPI antibodies were raised in rabbit against synthetic apoLTP peptides representing amino acid residues 375 – 394 (VFDHTPAVKPSHDEIKAARE), and 4326 – 4345 (DSFNNWDWGDEPESETNVMS). Anti-Cv-d was raised in guinea pig against a synthetic Cv-d peptide representing amino acid residues 1368 – 1387 (DQTPHNVRKKPYQWPIETSD). Anti-MTP was raised in rabbits against a peptide representing amino acid residues 82 – 342.

**Hemolymph Preparation**
Diluted hemolymph was obtained by bleeding larvae with fine forceps in ice-cold PBS. Hemocytes were removed by centrifugation for 30 min at 1500 g, 4 °C. To remove cellular fragments, the supernatant was subsequently spun for 30 min at 16 000 g, 4 °C. The supernatant from the second centrifugation step was snap-frozen in liquid nitrogen and stored at – 80 °C. For immunoblots, 5 µg protein were loaded on each lane. For density gradients, 200 µg protein were loaded on one gradient.

**Isopycnic Centrifugation in KBr Gradients**
Isopycnic centrifugation in KBr gradients was essentially performed according to [[5](#_ENREF_5)]. Briefly, hemolymph containing 200 µg protein was diluted to 100 µl with PBS, mixed with 900 µl of 0.5 mg/ml KBr and overlaid with 800 µl of 0.9 % NaCl. Gradients were then subjected to centrifugation at 259,000 g for 14 h at 4 °C, using an Optima MAX Ultracentrifuge and a TLS-55 rotor (Beckman Coulter). Subsequently, gradients were aliquoted into 100 µl fractions, protein precipitated with chloroform / methanol and subjected to immunoblotting.

**Isopycnic Centrifugation in Optiprep Gradients**
80 µl hemolymph was adjusted to 400 µl 50 % Optiprep (Axis-Shield). The sample was overlaid with 45 %, 35 % and 25 % Optiprep, 400 µl each, and 200 µl 10 % Optiprep. The gradient was then subjected to centrifugation at 285,000 g for 16 h at 4 °C, using an Optima MAX Ultracentrifuge and a TLS-55 rotor (Beckman Coulter). After centrifugation, the gradient was aliquoted into 100 µl fractions, protein precipitated and subjected to immunoblotting.

***Ex vivo* Lipid Transfer Experiments with Explanted Guts**
Hemolymph was prepared from feeding late third instar LTP RNAi larvae raised on yeast food or lipid-depleted food, and diluted in Grace’s insect medium at a final concentration of 4 – 5 µg / µl. Guts were dissected from early 3rd instar LTP RNAi larvae. Explanted guts were incubated for 4 h at 25 °C with the different hemolymph preparations. After incubation, hemolymph was recovered and further analyzed by isopycnic centrifugation as described above.

***Ex vivo* Lipid Transfer Experiments with Explanted Fat Bodies**

To distinguish Lpp that might be secreted by fat bodies from exogenously added Lpp, we utilized a variant harboring an N-terminal HA tag (HA-Lpp). Dense HA-Lpp was produced in the fat body with adh-GAL4, and LTP RNAi expressed under control of the same driver. Hemolymph was then collected in Grace’s insect medium and incubated with explanted fat bodies from third instar LTP RNAi or control larvae for 4 h at 25 °C. Subsequently, hemolymph was recovered and HA-Lpp density analyzed in isopycnic KBr gradients.

**Nile Red Staining**

Larval tissues were stained with Nile red according to [[6](#_ENREF_6)]. Briefly, samples were dissected in 1 µg/ml Nile red (Molecular Probes) in PBS, mounted in the same solution, and imaged immediately. Phospholipids were visualized by excitation of Nile red at a wavelength of 488 nm and fluorescence detection between 530 – 600 nm. Neutral lipid droplets were visualized by excitation of Nile red at a wavelength of 543 nm and fluorescence detection at > 635 nm.

**Filipin Staining**

Larval tissues were stained with filipin essentially according to [[7](#_ENREF_7)]. Briefly, samples were fixed with 4 % paraformaldehyde in PBS for 20 min. After several brief washes in PBS, samples were stained with 50 µg/ml filipin (Sigma-Aldrich) in PBS for 30 min. After several brief washes in PBS, samples were mounted in VECTASHIELD mounting medium (Vector Laboratories) and imaged immediately.

**Co-Staining of Lipid Droplets and Lipoproteins**

Tissues were stained as described in [[4](#_ENREF_4),[8](#_ENREF_8)]. Detergent was removed before incubation with secondary antibodies. To visualize lipid droplets, BODIPY 493/503 (Invitrogen) was added to the secondary antibodies in a concentration of 5 µg/ml.

**Reverse Transcription Polymerase Chain Reaction**

cDNA was synthesized from dissected fat bodies and guts with the RNeasy Mini Kit (Qiagen) according to manufacturer’s instructions. For each gene, primers were designed to span a small intron. PCRs were performed according to standard protocols with equivalent amounts of cDNA from each tissue, and as controls genomic DNA and H_2_O.

**Shotgun Lipidomics Mass Spectrometry**
Lipids from hemolymph and tissue homogenates were extracted and analyzed by shotgun lipidomics essentially according to [[9](#_ENREF_9)]. Briefly, samples were dissolved in 200 µl H2O; sample amounts were adjusted so that approximately 2 nmol of total lipid was extracted from each sample. The amount of hemolymph was adjusted by protein, the amount of tissue by number of organs.
To quantify lipids, internal standards were added to the samples prior to extraction: PC 18:3-18:3, 40 pmol; PE 17:0-17:0, 52 pmol; Cer 35:1;2, 20 pmol; Galactosyl Cer 30:1;2, 20 pmol; Lactosyl Cer 30:1;2, 20 pmol; Chol(D7), 50 pmol . Samples were then extracted with 1 ml chloroform-methanol [2.75:1 (v/v)] for 1 h at 4 °C. Samples were centrifuged for 2 min at 1000 g to promote phase separation; the lower organic phase (total lipid extract) was collected and evaporated.
Lipid extracts were resuspended in 100 μl chloroform-methanol [1:2 (v/v)] and were infused with a TriVersa NanoMate robotic nanoflow ion source (Advion Biosciences, Inc.). Quantitative lipid analysis was performed in positive ion mode on a LTQ-Orbitrap instrument (Thermo Fisher Scientific) as described previously [[9](#_ENREF_9)]. We excluded negatively charged lipid classes from analysis (phosphatidic acid, phosphatidylinositol phosphatidylserine), which represent only 1.5 % of total hemolymph lipid (JS, MC and AS, unpublished data). Sterols were derivatized according to [[10](#_ENREF_10)], and analyzed on an LTQ-Orbitrap. CerPE species were quantified using the PE 17:0-17:0 internal standard. Lipid identification was performed using LipidXplorer software [[11](#_ENREF_11)].

Unless stated otherwise, hemolymph lipids were normalized per protein, and cellular acylglycerols per polar lipid (see also Figure S11).

**Supplemental References**

1. Ejsmont RK, Sarov M, Winkler S, Lipinski KA, Tomancak P (2009) A toolkit for high-throughput, cross-species gene engineering in Drosophila. Nat Methods 6: 435-437.

2. Markstein M, Pitsouli C, Villalta C, Celniker SE, Perrimon N (2008) Exploiting position effects and the gypsy retrovirus insulator to engineer precisely expressed transgenes. Nat Genet 40: 476-483.

3. Marois E, Eaton S (2007) RNAi in the Hedgehog signaling pathway: pFRiPE, a vector for temporally and spatially controlled RNAi in Drosophila. Methods Mol Biol 397: 115-128.

4. Panakova D, Sprong H, Marois E, Thiele C, Eaton S (2005) Lipoprotein particles are required for Hedgehog and Wingless signalling. Nature 435: 58-65.

5. Shapiro JP, Law JH (1983) Locust adipokinetic hormone stimulates lipid mobilization in Manduca sexta. Biochem Biophys Res Commun 115: 924-931.

6. Greenspan P, Mayer EP, Fowler SD (1985) Nile red: a selective fluorescent stain for intracellular lipid droplets. J Cell Biol 100: 965-973.

7. Voght SP, Fluegel ML, Andrews LA, Pallanck LJ (2007) Drosophila NPC1b promotes an early step in sterol absorption from the midgut epithelium. Cell Metab 5: 195-205.

8. Eugster C, Panakova D, Mahmoud A, Eaton S (2007) Lipoprotein-heparan sulfate interactions in the Hh pathway. Dev Cell 13: 57-71.

9. Carvalho M, Schwudke D, Sampaio JL, Palm W, Riezman I, et al. (2010) Survival strategies of a sterol auxotroph. Development 137: 3675-3685.

10. Sandhoff R, Brugger B, Jeckel D, Lehmann WD, Wieland FT (1999) Determination of cholesterol at the low picomole level by nano-electrospray ionization tandem mass spectrometry. J Lipid Res 40: 126-132.

11. Herzog R, Schwudke D, Schuhmann K, Sampaio JL, Bornstein SR, et al. (2011) A novel informatics concept for high-throughput shotgun lipidomics based on the molecular fragmentation query language. Genome Biol 12: R8.
